# Polyzwitterionic hydrogel electrolytes based on imidazolium cations and sulfonate anions enable stable Zn–I_2_ batteries by regulating Zn deposition and inhibiting polyiodide shuttle

**DOI:** 10.1039/d6sc03860c

**Published:** 2026-06-26

**Authors:** Bokang Yuan, Fangfei Liu, Yufan Lei, Minghui Xu, Yuting Xie, Huaren Fan, Feng Zeng, Xiong Liu

**Affiliations:** a State Key Laboratory of Chemistry and Utilization of Carbon Based Energy Resources, College of Chemistry, Xinjiang University Urumqi 830017 Xinjiang PR China liufangfei1214@163.com liuxiong@xju.edu.cn

## Abstract

Aqueous zinc–iodine batteries represent a promising candidate for new energy storage systems owing to their abundant resources and high energy density. However, the challenges associated with the polyiodide shuttle and zinc dendrite growth are the critical barriers to the large-scale energy storage system. Herein, polyzwitterionic hydrogel electrolytes (PMVP) are prepared from imidazolium cation monomers and sulfonate anion monomers for regulating dendrite growth and polyiodide shuttle. The sulfate anions restrict the diffusion of iodide ions through strong electrostatic repulsion while capturing free water molecules *via* strong coordination. Meanwhile, the imidazolium cations guide the uniform deposition of zinc ions to inhibit the dendrite formation and intercept those iodide ions that bypass the electrostatic repulsion barrier. This hydrogel electrolyte shows high ionic conductivity (33.89 mS cm^−1^) and transference number (0.74). The Zn//PMVP//Zn symmetric cell cycles stably over 4500 h at 1 mA cm^−2^/1 mA h cm^−2^ and 1000 h at 10 mA cm^−2^/1 mA h cm^−2^. The capacity retention of the PMVP-based Zn//I_2_ battery reaches 71.48% after 10 000 cycles (1.0 A g^−1^), and the flexible pouch cell maintains stable operation under extreme conditions. These cation–anion synergistic polyzwitterionic hydrogel electrolytes provide a novel strategy for addressing dendrite growth and polyiodide shuttle issues in zinc–iodine batteries.

## Abstract 2

A polyzwitterionic hydrogel electrolyte based on an imidazolium cation and sulfonate anion regulates the zinc anode deposition and iodine cathode shuttle behaviors in zinc–iodine batteries.

## Graphical abstract

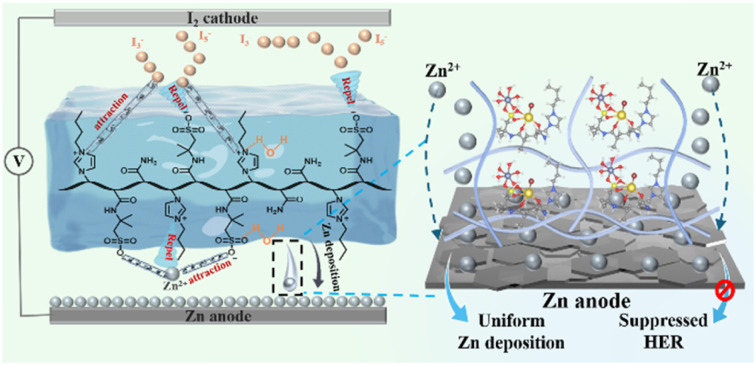

## 1. Introduction

The energy shortage has accelerated the rapid advancement of safer, more efficient renewable energy storage systems. Aqueous zinc-ion batteries (AZIBs) have become a vital energy storage device because of their high safety, abundant raw material resources and high energy density.^1^ Among various AZIBs, zinc–iodine batteries have become a research hotspot due to their outstanding resource advantages, moderate specific capacity (211 mA h g^−1^), and reasonable discharge voltage plateau (1.38 V).^2^ However, they are constrained by inherent limitations: uneven zinc deposition on the zinc anode leads to the formation of dendrites,^3^ thereby increasing the risk of internal short circuits and lowering the energy density.^4^ The polyiodides (I_3_^−^/I_5_^−^) generated by the iodine cathode trigger severe shuttling effects, resulting in self-discharge and overcharge behavior.^5,6^ Furthermore, these polyiodide ions react with the zinc anode, accelerating corrosion and causing active material loss. To address these issues, researchers have employed various approaches, including improvements to iodine-loading materials, the use of novel separators,^7,8^ and modifications to the electrolyte. Among these methods, the development of a novel electrolyte is key to overcoming this bottleneck. In conventional liquid electrolytes, side reactions like zinc dendrite growth and zinc corrosion greatly weaken the cycle lifespan and coulombic efficiency of zinc–iodine batteries.^9^ Solid electrolytes with high mechanical hardness lead to poor interfacial wettability with electrodes, and the high interfacial impedance limits the electrochemical performance of zinc–iodine batteries.^10^ Therefore, the design of new-type electrolytes to concurrently regulate Zn deposition and inhibit polyiodide shuttle is still challenging in zinc–iodine batteries.

As a “bridge” connecting the advantages of liquid and solid electrolytes, hydrogel electrolytes confine electrolytes within a polymer three-dimensional network, integrating the functions of leakage resistance, ion transport channel construction, and zinc dendrite growth suppression.^11^ Due to the inherent flexibility, hydrogel electrolytes effectively dissipate mechanical stress, alleviating structural damage and performance decay to ensure the long-range reliability of energy storage devices. Through reasonable molecular engineering, hydrogel electrolytes can achieve accurate control over solvation configuration and ion transport behavior, thus boosting electrochemical storage ability. Polyelectrolyte hydrogels with ionic groups on polymer chains are particularly attractive for zinc–iodine batteries because of their adjustable ionic interactions and excellent ion transport ability.^12,13^ Based on the charge properties of functional groups, polyelectrolyte hydrogels are divided into polyanionic, polycationic, and polyzwitterionic hydrogels.^14,15^ Polyanionic hydrogels (generally including sulfonate or carboxylate groups) can block polyiodide shuttle *via* electrostatic repulsion and suppress side reactions by coordinating free water^16,17^ but lack the ability to guide uniform Zn^2+^ deposition, thus failing to inhibit dendrite growth.^18^ Polycationic hydrogels (generally including quaternary ammonium or imidazolium groups) can realize electrostatic shielding of the zinc anode to suppress dendrites^19,20^ but show weak inhibition on polyiodide shuttle due to the absence of the electrostatic repulsion effect, leading to poor capacity retention.^21,22^ Polyzwitterionic hydrogels simultaneously containing anionic and cationic groups are capable of forming independent anion/cation transfer channels to facilitate the ion transport kinetics; meanwhile, the anionic and cationic groups with intrinsic hydrophilicity can coordinate with water molecules to suppress the hydrogen evolution reaction (HER). It is important to note that the anions with negative charges in the polyzwitterionic hydrogels have the ability to generate electrostatic repulsion toward polyiodides, resulting in the suppression of polyiodide shuttle.^23–25^ Notwithstanding some significant progress, a lot of efforts are still required to engineer the molecular structure and charge composition of polyzwitterionic hydrogel electrolytes for solving the challenges of zinc dendrite growth, polyiodide shuttle and side reactions, eventually enhancing the electrochemical storage performance of zinc–iodine batteries.

In this work, a polyzwitterionic hydrogel electrolyte (PMVP) was fabricated by one-pot free-radical copolymerization of acrylamide (AM), 1-butyl-3-vinylimidazolium bromide (VBIMBr, cationic monomer) and 2-acrylamido-2-methylpropanesulfonic acid sodium salt (NaAMPS, anionic monomer) to adjust the zinc anode deposition and iodine cathode shuttle behaviors in zinc–iodine batteries. In this system, the anionic sulfonate groups restrict polyiodide diffusion *via* electrostatic repulsion and trap free water to suppress side reactions, while the cationic imidazole groups guide uniform Zn^2+^ deposition to inhibit dendrites and intercept polyiodides that bypass the electrostatic repulsion barrier. The dual cation–anion confinement effect realizes the efficient and simultaneous suppression of zinc dendrite growth and polyiodide shuttle. The prepared PMVP hydrogel electrolyte exhibited high ionic conductivity and Zn^2+^ transference number. The Zn//PMVP//Zn symmetric cell showed good stability over 4500 h (1 mA cm^−2^/1 mA h cm^−2^) and for 1000 h (10 mA cm^−2^/1 mA h cm^−2^). The Zn//PMVP//I_2_ cell exhibited a capacity retention of 71.48% and an average coulombic efficiency of 99.50% at 1.0 A g^−1^. This strategy provides a technical solution for design of high-performance hydrogel electrolytes to optimize the zinc anode deposition and iodine cathode shuttle in zinc–iodine batteries.

## 2. Results and discussion

### 2.1. Preparation and characterization

**Fig. 1:**
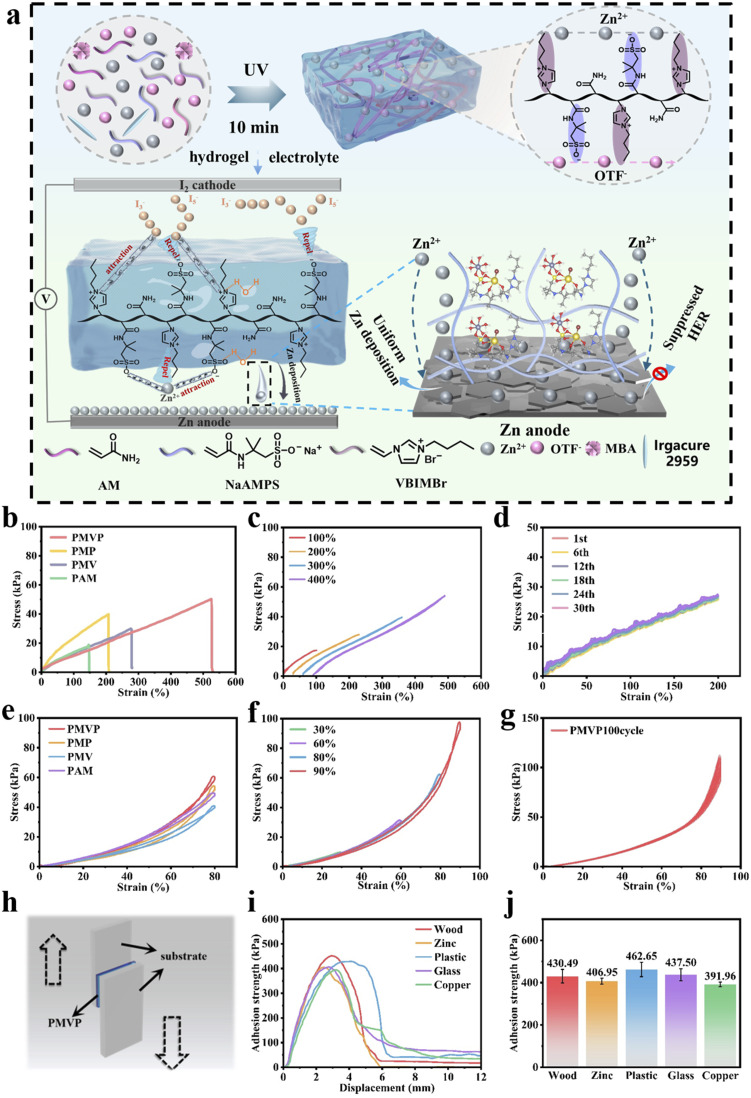
(a) Diagram of the preparation of PMVP hydrogel and its work mechanism in zinc–iodine batteries. (b) Stress–strain profiles. (c) Cyclic tensile curves of PMVP under different strains. (d) Cyclic tensile test of PMVP hydrogel at 200% strain for 30 cycles. (e) Compressive comparison curves of hydrogel. (f) Cyclic compressive curves of PMVP under different strains. (g) Cyclic compressive test of PMVP at 90% strain for 100 cycles. (h) Illustration of the lap shear test. (i) Adhesion curves of PMVP. (j) Adhesion strength of PMVP.

Fig. 1a and S1 illustrate that the PMVP hydrogel electrolyte was prepared *via* a one-pot free-radical copolymerization strategy. Among the components employed in the process, AM constructed the polymer backbone and endowed the electrolyte with basic mechanical properties and structural flexibility; NaAMPS introduced sulfonate anion (–SO_3_^−^) groups, which built ion transport channels to facilitate the rapid migration of Zn^2+^; VBIMBr induced uniform zinc deposition *via* the electrostatic shielding effect, and its imidazole groups facilitated electrostatic interactions with –SO_3_^−^ to enhance the mechanical performance.^23^ Furthermore, the –SO_3_^−^ groups exerted electrostatic repulsion on negatively charged polyiodides (I_3_^−^/I_5_^−^), while the imidazole groups actively intercepted polyiodides that bypassed the –SO_3_^−^ repulsion barrier through electrostatic attraction, thus remarkably suppressing the polyiodide shuttle effect.^26^ Fig. S2 reveals that the PMVP hydrogel electrolyte possessed uniform porous channels, which facilitated the transport of water molecules and Zn^2+^. Contrarily, these porous structures cannot accelerate the diffusion of large-sized polyiodides, owing to the combined effect of physical steric hindrance and electrostatic immobilization. EDS elemental mapping results demonstrated the uniform distribution of C, N, O, F, S, Zn, Br, and Na within the PMVP hydrogel (Fig. S3). As illustrated in Fig. S4, the stretching vibration of C=O was observed at 1662 cm^−1^, and the N–H stretching and bending vibrations were found at 3440 and 1608 cm^−1^, respectively; these features were in good agreement with the molecular structure of polyacrylamide (PAM). The stretching vibrations of –CF_3_ in symmetric and asymmetric modes were observed at 1255 and 1174 cm^−1^, respectively, the symmetric stretching vibration of –SO_3_^−^ was found at 1029 cm^−1^, and the asymmetric bending vibration of –SO_3_CF_3_– was discovered at 642 cm^−1^. Additionally, a distinct characteristic peak at 1030 cm^−1^ corresponded to the planar vibration of –SO_3_Na. Notably, C=C absorption characteristics were not identified in PMVP at 990 cm^−1^ and 820 cm^−1^, nor was an obvious C=C stretching vibration peak observed at 1635 cm^−1^, confirming the completion of the free radical polymerization reaction. We further conducted XPS analysis to confirm the chemical bonding states of the PMVP hydrogel (Fig. S5). The C 1s spectrum exhibited four peaks, assigned to C–C (284.8 eV), C–N/C–S (286.3 eV), C=O (288.8 eV), and C–F (292.8 eV). The N 1s spectrum displayed two peaks: 400.2 eV for C–N bonds and 401.6 eV for N–H bonds, which were derived from the nitrogen-containing groups in VBIMBr and AM. The Zn spectrum exhibited distinct characteristic peaks at 1022.9 and 1045.9 eV, typical of Zn^2+^ in the hydrogel. Collectively, these characterization results confirmed that the PMVP hydrogel electrolyte was successfully synthesized.

For the hydrogel electrolyte to be applied in energy storage devices, its mechanical performance is of great significance and cannot be ignored, as it prevented the hydrogel from deforming and even being damaged under external stress.^27^ As depicted in Fig. 1b, stress–strain curves were used to evaluate tensile properties. Benefiting from the electrostatic interaction effect of cations and anions, the PMVP hydrogel exhibited significantly higher elongation and tensile strength (525.57%, 50.28 kPa) than poly(acrylamide-*co*-2-acrylamido-2-methyl-1-propanesulfonate) (PMP) (207.11%, 30.99 kPa), poly(acrylamide-*co*-1-butyl-3-vinylimidazolium bromide) (PMV) (276.07%, 31.46 kPa), and polyacrylamide (PAM) (146.97%, 18.63 kPa). Moreover, the PMVP hydrogel could recover to its initial shape even when stretched to strains between 100% and 400% (Fig. 1c). At 200% strain, the stress–strain hysteresis curves of PMVP almost completely overlapped (Fig. 1d), demonstrating superior fatigue resistance. As shown in Fig. S6, the PMVP hydrogel maintained its structural integrity and recovered its original shape after multiple twists and knotting, further confirming its excellent elastic properties. The compressive performance of the PMVP hydrogel was also evaluated. At the same compressive strain of 80%, PMVP (58.83 kPa) exhibited higher compressive stress than PMP (50.17 kPa), PMV (35.04 kPa), and PAM (40.70 kPa), indicating that PMVP could withstand greater loads and possessed superior compressive resistance (Fig. 1e). Furthermore, the PMVP hydrogel could return to its original state after compression under strains from 30% to 90% (Fig. 1f). Even after 100 compression cycles at 90% strain, only a small hysteresis was observed. Upon stress removal, the PMVP hydrogel fully recovered with almost overlapping curves (Fig. 1g), confirming its robust compression resistance. Overall, the PMVP hydrogel exhibited excellent durability and structural stability during repeated stretching and compression cycles, which provided strong support for its practical use in energy storage systems.

Good interfacial adhesion with the substrates is essential for reducing the contact resistance, so the adhesive properties of the PMVP hydrogel were investigated. In PMVP, hydrogen bonds and electrostatic interactions effectively enhanced the interfacial bonding between the hydrogel and a wide range of substrates. Lap shear tests were performed to characterize the adhesive behavior (Fig. 1h), and the adhesion curves of PMVP on different substrates were measured (Fig. 1i). The adhesion strength of the PMVP hydrogel was determined to be 430.49 kPa (wood), 406.95 kPa (zinc foil), 462.65 kPa (plastic), 437.50 kPa (glass), and 391.96 kPa (copper foil) (Fig. 1j). As displayed in Fig. S7, the PMVP hydrogel possessed robust adhesive ability toward all the substrates under investigation. This excellent adhesion property was expected to effectively reduce the interfacial resistance and improve the interfacial stability, thereby enhancing the overall performance of energy storage devices assembled with this hydrogel electrolyte.

### 2.2. Electrochemical properties

**Fig. 2:**
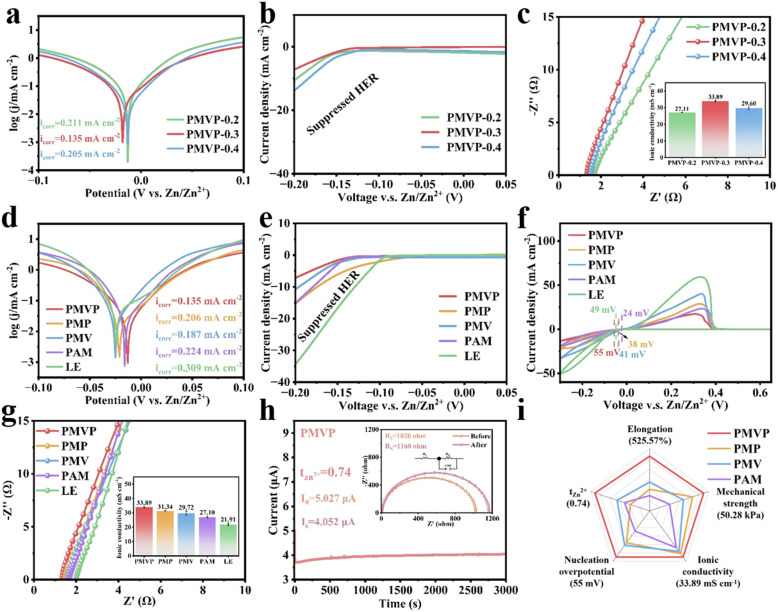
(a) Tafel plots of PMVP electrolytes with different variables. (b) LSV curves of PMVP electrolytes with different variables. (c) Nyquist plots of PMVP electrolytes with different variables (inset: ionic conductivity). (d) Tafel plots of PMVP, PMP, PMV, PAM, and liquid electrolytes. (e) LSV profiles acquired for various electrolyte systems. (f) CV profiles obtained from different electrolyte systems. (g) Nyquist plots for various electrolyte systems (inset: ionic conductivity). (h) *I*–*t* curves .(inset: electrochemical impedance spectroscopy (EIS) curves). (i) Overall performance comparison chart.

To determine the ability of hydrogel electrolytes to inhibit dendrite growth, their electrochemical behaviors were key evaluation parameters. To assess the electrochemical stability, symmetric Zn//Zn batteries were fabricated. The contents of NaAMPS and VBIMBr in PMVP were optimized, and their molar ratio of VBIMBr to NaAMPS was maintained at 1 : 1. The NaAMPS content was set at 0.2 g (PMVP-0.2), 0.3 g (PMVP-0.3), and 0.4 g (PMVP-0.4). It was found that PMVP-0.3 exhibited the lowest corrosion current density (0.135 mA cm^−2^, Fig. 2a), the most effective HER inhibition effect (Fig. 2b), and the highest ionic conductivity (33.89 mS cm^−1^) (Fig. 2c). So, the electrostatic cross-linking between anions (–SO_3_^−^) and cations (imidazole groups) of PMVP-0.3 reached an optimal state. This not only provided sufficient network strength and stability but also avoided hydrogel network blockage and reduction in the ionic conductivity caused by excessive crosslinking density. Moreover, PMVP-0.3 minimized water activity *via* coordination, resulting in the strongest HER inhibition and fewer corrosion side reactions. Fig. S8 exhibits that PMVP-0.3 possessed superior ability to suppress the uncontrolled growth of zinc dendrites. Given these distinct advantages, PMVP-0.3 was selected as the preferred hydrogel electrolyte for all subsequent electrochemical characterization studies.

Fig. 2d presents the corrosion current density of different electrolytes, in which the PMVP hydrogel electrolyte exhibited the lowest value of 0.135 mA cm^−2^. In comparison, the corrosion current densities of PMP, PMV, PAM, and liquid electrolyte (LE) (a glass fiber separator with 1 M Zn(OTf)_2_) were 0.206, 0.187, 0.224, and 0.309 mA cm^−2^, respectively. This confirmed that the PMVP hydrogel electrolyte showed a better corrosion resistance. Fig. 2e exhibits that the PMVP hydrogel also had a wider electrochemical window, enabling the effective inhibition of the HER. This was because the –SO_3_^−^ groups had a strong coordinating capacity with Zn^2+^, meanwhile the imidazolium cations separated water from the zinc anode *via* electrostatic repulsion. Fig. 2f presents the CV curves of Zn//Cu batteries, indicating that the nucleation overpotential of PMVP (55 mV) was higher than that of LE (49 mV), PMP (38 mV), PMV (41 mV), and PAM (24 mV). This demonstrated that PMVP electrolyte accelerated the nucleation kinetics and facilitated the generation of finer zinc electrodeposits.^28^ Additionally, the ionic conductivity was measured by EIS, as illustrated in Fig. 2g. PMVP showed an ionic conductivity of 33.89 mS cm^−1^, which was significantly greater than that of PMP (31.34 mS cm^−1^), PMV (29.72 mS cm^−1^), PAM (27.10 mS cm^−1^), and LE (21.91 mS cm^−1^). This indicated that the –SO_3_^−^ groups of NaAMPS and imidazolium cations of VBIMBr effectively enhanced the ionic conductivity. Fig. 2h and S9 show that the Zn^2+^ transference number (*t*_Zn^2+^_) of PMVP was 0.74, which was more than that of PMP (0.26), PMV (0.44), PAM (0.34), and LE (0.23). The imidazolium cations immobilized OTF^−^ anions, increasing the Zn^2+^ transference number of PMVP. Meanwhile, the –SO_3_^−^ groups anchored to the polymer backbone provided ion transport channels along the polymer chains for positively charged Zn^2+^ through electrostatic attraction and coordination, thereby further improving the Zn^2+^ transference number.^29,30^ This also helped to significantly reduce the desolvation energy barrier, implying a more efficient charge transfer mechanism and further mitigating dendrite growth.^31^ Furthermore, to confirm that PMVP's superior performance stems from the copolymerized anionic and cationic groups rather than simple physical mixing, PMVP was compared with MVP (a physical mixture of hydrogel components). As shown in Fig. S10, compared to MVP, PMVP exhibits lower corrosion current density, more suppressed HER activity, lower Zn nucleation overpotential, and higher ionic conductivity. These results indicate that the copolymerized amphoteric network can more effectively regulate Zn^2+^ transport and side reactions. Fig. 2i shows the comprehensive advantages of the PMVP hydrogel electrolyte. It has outstanding mechanical properties, high elongation, high nucleation overpotential, and exceptional ionic conductivity. Thus, it shows great potential for use as an electrolyte in flexible AZIBs.

### 2.3. Solvation chemistry

**Fig. 3:**
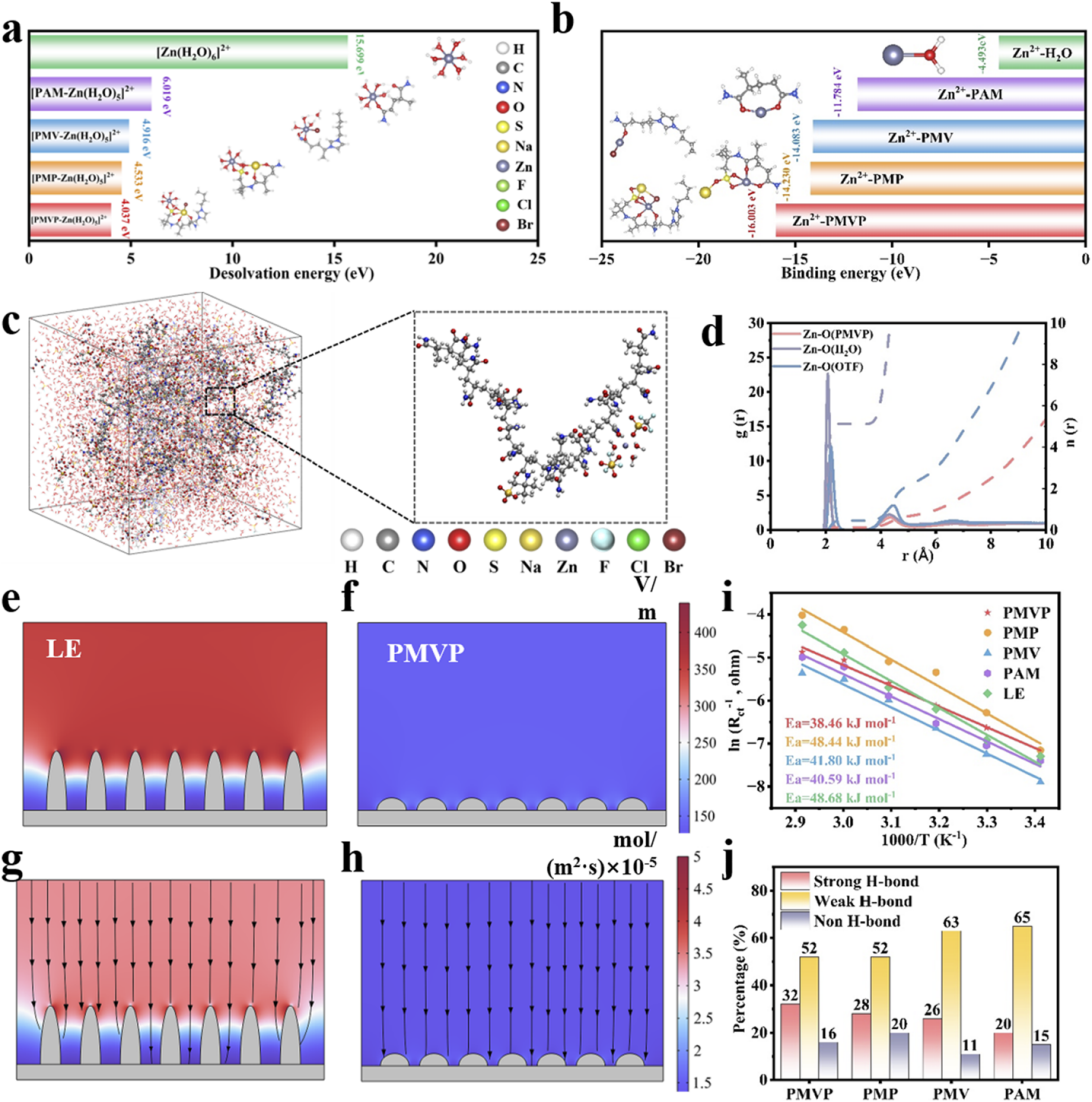
(a) Desolvation energies. (b) Binding energies. (c) MD simulation of Zn^2+^ ions in PMVP. (d) RDF of PMVP. (e) Zinc anode electric field in LE. (f) Zinc anode electric field in PMVP. (g) Flux of Zn^2+^ in LE. (h) Flux of Zn^2+^ in PMVP. (i) Arrhenius plot and activation energy (*E*_a_). (j) Relative proportions of hydrogen bonds.

The solvation structure plays an important function in inhibiting dendrite growth.^32^ To explore the solvation chemistry, we carried out DFT calculations. Desolvation energy represents the energy needed for Zn^2+^ to break away from the solvation shell composed of adjacent water molecules. A lower value indicated that Zn^2+^ could more easily break away from the solvation shell, leading to higher kinetic efficiency for migration in the electrolyte and participation in electrode reactions. Conversely, a higher value made the desolvation process more difficult, which became an “energy barrier” for the reactions in zinc-ion batteries.^30^ As shown in Fig. 3a, theoretical calculation analysis revealed that PMVP, PMP, PMV, and PAM hydrogels coordinated with Zn^2+^ to replace water molecules, reducing the desolvation energy of Zn^2+^ from 15.699 eV ([Zn(H_2_O)_6_]^2+^) to 4.037 eV, 4.53 eV, 4.916 eV, and 6.019 eV, respectively. Owing to the synergistic coordination effect of anionic and cationic monomers, PMVP exhibited stronger ability to decompose the hydration shell of Zn^2+^ compared to PMP and PMV, resulting in the lowest desolvation energy.^33^ This modulated the solvation structure, thus improving the ion transport channel.^34^ As illustrated in Fig. 3b, PMP (−14.230 eV) with –SO_3_^−^ anionic groups and PMV (−14.083 eV) with lone-pair-containing imidazole groups preferentially captured Zn^2+^, leading to significantly lower binding energies than that between H_2_O and Zn^2+^ (−4.493 eV) and between the acyl group of PAM and Zn^2+^ (−11.784 eV). Furthermore, through synergistic effects, the polyzwitterionic PMVP hydrogel, containing –SO_3_^−^ and imidazole groups simultaneously, exhibited a further reduced binding energy of −16.003 eV with Zn^2+^,^35^ thus reconstructing the solvation structure and suppressing dendrite growth simultaneously.^36^

MD simulations were carried out based on the LE and PMVP models (detailed in Tables S1 and S2), as shown in Fig. 3c and S11. The results verified that PMVP replaced water molecules in the Zn^2+^ coordination shell. This process formed a distinctive solvation structure.^37^ Radial distribution function (RDF) analysis (Fig. 3d and S12) showed that at the same Zn–O coordination distance of 2 Å, the number of coordinated water moieties around Zn^2+^ in PMVP (5.115) was significantly less than that in LE (5.636). This revealed that PMVP effectively regulated the solvation structure, the hydration shell of Zn^2+^ is partially destroyed by the hydrogel network, and the partially dehydrated Zn^2+^ exhibits lower migration resistance during transportation, which are conducive to accelerating ion movement and forming fast ion transport pathways. To further verify that PMVP modified the solvation structure, finite element simulation tests were conducted. As shown in Fig. 3e and f, alternating blue-red rate fluctuation peaks emerged near the zinc electrode surface in the LE system. This phenomenon was due to obvious charge accumulation on dendrite tips in the LE, further leading to the “tip effect”. In contrast, the PMVP electrolyte system exhibited a uniformly blue tone with no obvious rate fluctuations. This was because the polymer network of PMVP had a stronger coordination interaction effect with Zn^2+^, and its regular network structure enabled more uniform transport of Zn^2+^ at the interface, significantly modulating the solvation structure of Zn^2+^. Zn^2+^ flux analysis (Fig. 3g and h) further verified the differences between the two systems. In LE, vertically downward transport arrows were densely distributed, but the rate fluctuation near the electrode interface easily caused local concentration polarization of Zn^2+^, which induced dendrite growth. In PMVP, the transport arrows were uniformly distributed, and its homogeneous transport characteristic could effectively suppress the Zn^2+^ concentration at the zinc anode, thereby alleviating dendrite growth. Further reaction kinetic tests were performed on PMVP. As shown in Fig. 3i and S13, the activation energy (*E*_a_) of PMVP (38.46 kJ mol^−1^) was markedly smaller than that of PMP (48.44 kJ mol^−1^), PMV (41.80 kJ mol^−1^), PAM (40.59 kJ mol^−1^), and LE (48.68 kJ mol^−1^). This confirmed that PMVP could significantly reduce the energy required for the desolvation process, accelerate desolvation, and effectively inhibit dendrite formation. The interactions between water molecules were studied using Raman spectroscopy (Fig. 3j and S14). This revealed that the proportion of strong hydrogen bonds in PMVP was 32%, which is higher than that in PMP (28%), PMV (26%), and PAM (20%). This indicated that active water was locked in the PMVP electrolyte, significantly inhibiting water-induced side reactions.^38,39^

### 2.4. Zinc deposition behavior

**Fig. 4:**
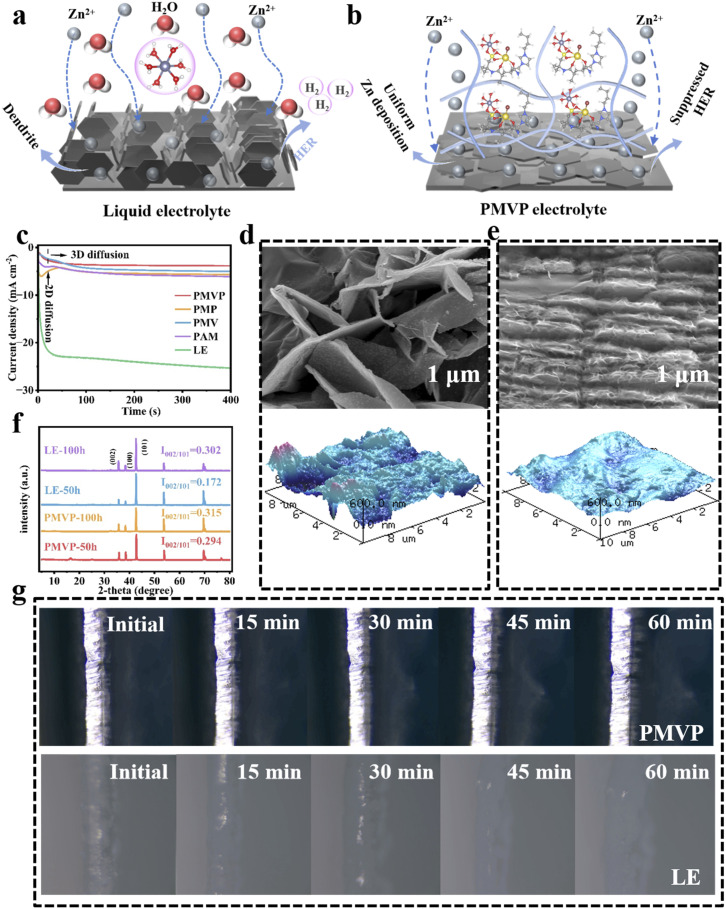
(a) Illustration of zinc deposition in LE. (b) Illustration of zinc deposition in PMVP electrolyte. (c) CA curves of PMVP, PMP, PMV, and liquid electrolytes. (d and e) SEM and AFM images of zinc anodes. (f) XRD of zinc anodes. (g) *In situ* optical microscopy images.

Uneven Zn deposition tended to trigger dendrite growth and the tip effect, which harmed the overall performance of zinc-ion batteries. So, the zinc deposition behavior in hydrogel electrolytes was systematically investigated. As illustrated in Fig. 4a, the LE was unavoidably in intimate contact with water moieties, triggering intense HER and causing the tip effect of zinc dendrites. In comparison, the sulfonate anions (–SO_3_^−^) effectively guided the uniform transport of Zn^2+^ (Fig. 4b). The imidazolium cation groups promoted uniform zinc deposition through the charge shielding effect. The anions and cations worked together to build ion transport channels and inhibited the zinc tip effect, further proving their synergistic regulation on zinc deposition, which was consistent with the solvation chemistry characteristics. CA measurements were performed to investigate Zn^2+^ deposition. In Fig. 4c, the current of the LE increased continuously for 400 s, indicating two-dimensional diffusion, and the zinc deposition was disordered. In comparison, PMVP transitioned smoothly to the three-dimensional diffusion stage within 25 s, which was faster than PMP, PMV, and PAM. This result proved that PMVP enabled more uniform zinc deposition. This was due to the ion transport pathways formed by anions and cations, as well as the solvation structure adjusted by synergistic coordination.^40^

After 100 h of cycling, the zinc anodes were examined using SEM and AFM. As presented in Fig. 4d, the zinc anode in LE exhibited a dendritic deposition morphology with interlaced protrusions, indicating the typical characteristics of zinc dendrite growth. The AFM results revealed significant surface height fluctuations and high roughness, reflecting the non-uniformity of zinc deposition in LE. Such a rough surface exacerbated the interfacial impedance, and the protruding dendritic sites tended to induce side reactions. In comparison, the zinc anode in PMVP displayed a dense, flat layered deposition morphology without obvious dendritic protrusions, only uniform wrinkled textures (Fig. 4e). This was due to the uniform migration and deposition of Zn^2+^*via* coordination with polymer functional groups, preventing zinc dendrite growth. The AFM results demonstrated that the surface height fluctuations of the zinc anode in PMVP were significantly reduced, with a smoother overall surface and uniform color distribution. A flat zinc deposition surface could reduce the inhomogeneity of interfacial electric field distribution, decrease side reactions, enhance the interfacial contact compatibility, and reduce interfacial impedance. Similar behavior was detected at 1 mA cm^−2^ and 1 mA h cm^−2^ for 50 h and 200 h and at 2 mA cm^−2^ and 2 mA h cm^−2^ for 100 h (Fig. S15 and S16). After zinc plating/stripping cycles for different durations, the crystal structure of the zinc anode surface was investigated *via* XRD (Fig. 4f). The peak intensity ratios of (002)/(101) for the zinc anodes in PMVP electrolyte were 0.294 and 0.315 after 50 h and 100 h cycling, respectively, which were markedly higher than those in the LE system (0.172 and 0.302). As cycling proceeded (Fig. S17), the peak intensity ratio was further improved. These results indicated that PMVP electrolyte facilitated the preferential deposition of Zn^2+^ on the (002) crystal plane, confirming the uniform zinc deposition during long-term cycling. *In situ* optical observation was adopted to track the deposition process of the zinc anode at 20 mA cm^−2^. As shown in Fig. 4g, the LE struggled to achieve uniform zinc deposition; non-uniform nucleation occurred on the anode surface in the beginning, accompanied by rapid dendrite growth. PMVP can precisely regulate zinc ion migration, enabling the zinc anode to maintain a flat morphology during prolonged deposition and demonstrating optimal dendrite suppression capability. Although the combined performance of PMP and PMV is slightly inferior to that of PMVP, the electrode surface remains relatively flat during the 60 minute observation period. In contrast, the dendrite suppression effect of PAM is significantly reduced, and the electrode surface gradually exhibits a trend toward uneven deposition and bubble formation (Fig. S18). These observations further demonstrated the uniform zinc deposition behavior and effective suppression of the HER, which were comprehensive results of PMVP electrolyte's optimized mechanical properties (mechanical barrier), solvation chemistry (low desolvation energy and water locking), and ion transport performance (uniform flux distribution).

### 2.5. Battery reversibility and cyclability

**Fig. 5:**
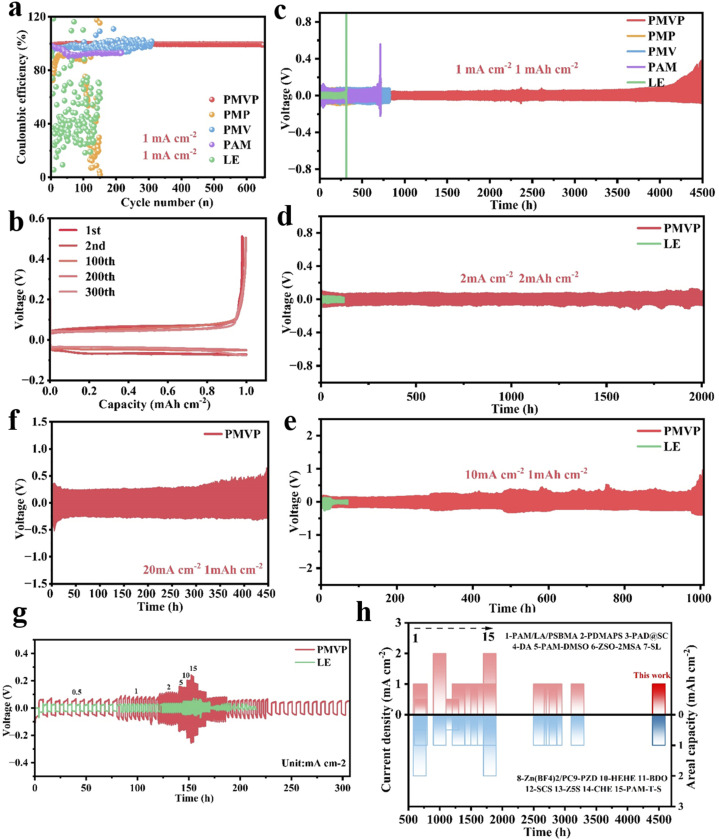
(a) CE of PMVP and liquid electrolytes. (b) Voltage distribution curves. (c–f) Cycling of Zn//Zn batteries. (g) Voltage distribution at different current densities. (h) Comparison of cycle lifespan with recently reported symmetric batteries.

To assess the performance of the zinc anode, its reversibility and cyclability were systematically analyzed. Fig. 5a shows that the Zn//PMVP//Cu half cells maintained stability for 600 cycles, with an average CE of 99.65%. In comparison, the Zn//Cu half cells with PMP, PMV, and liquid electrolytes experienced significant fluctuations and sharp declines within 100 cycles. To investigate the underlying mechanism of this phenomenon, CE testing was performed on the first cycle of Zn//Cu half-cells. A CE of 97.88% (PMVP) in the initial cycle was greater than that of PMP (85.11%), PMV (95.07%), PAM (97.72%), and liquid (93.96%) electrolytes (Fig. S19). This indicated that the PMVP-based half-cell stabilized more rapidly during the initial cycle. Moreover, the PMVP electrolyte half-cell maintained smooth voltage curves and consistent CE over 300 cycles (Fig. 5b), confirming its excellent initial stability and reversibility. The sustained stability of the Zn anode was assessed in Zn//Zn symmetric cells. At 1 mA cm^−2^ and 1 mA h cm^−2^, the PMVP cell exhibited sustained operation for 4500 h (Fig. 5c). By contrast, symmetric cells equipped with PMP, PMV, PAM, and liquid electrolytes experienced early failure within 800 h, owing to short circuits triggered by intensive dendrite propagation and severe hydrogen evolution reaction inside the cells. This outstanding electrochemical performance stemmed from the strong synergistic effect between anionic and cationic moieties in the PMVP electrolyte, which efficiently inhibited the dendrite formation and HER.^41^

The Zn//PMVP//Zn symmetric battery also delivered a high cycle life of 2000 h at 2 mA cm^−2^ and 2 mA h cm^−2^ (Fig. 5d). Furthermore, as shown in Fig. 5e and S20, the Zn//PMVP//Zn symmetric cell demonstrated prolonged cycling durability even at elevated current densities, operating continuously for 1100 h at 8 mA cm^−2^ and 1000 h at 10 mA cm^−2^. It maintained stable cycling at ultrahigh current densities of 20 and 30 mA cm^−2^ for 450 and 140 h, respectively (Fig. 5f and S21). These results confirmed that the Zn//PMVP//Zn cell possessed good cyclability and reversibility over a broad range of current densities. With the increase of current from 0.5 to 15 mA cm^−2^, the PMVP-based cell showed stability within 300 h (Fig. 5g), which further verified that the PMVP electrolyte suppressed dendrite growth and the HER under different current densities. In addition, the Zn//PMVP//Zn cell showed better cycling stability and longer lifespan than most recently reported symmetric systems (Fig. 5h and Table S3), revealing its application potential in aqueous zinc-ion battery devices.

### 2.6. Suppression of the polyiodide shuttle effect

**Fig. 6:**
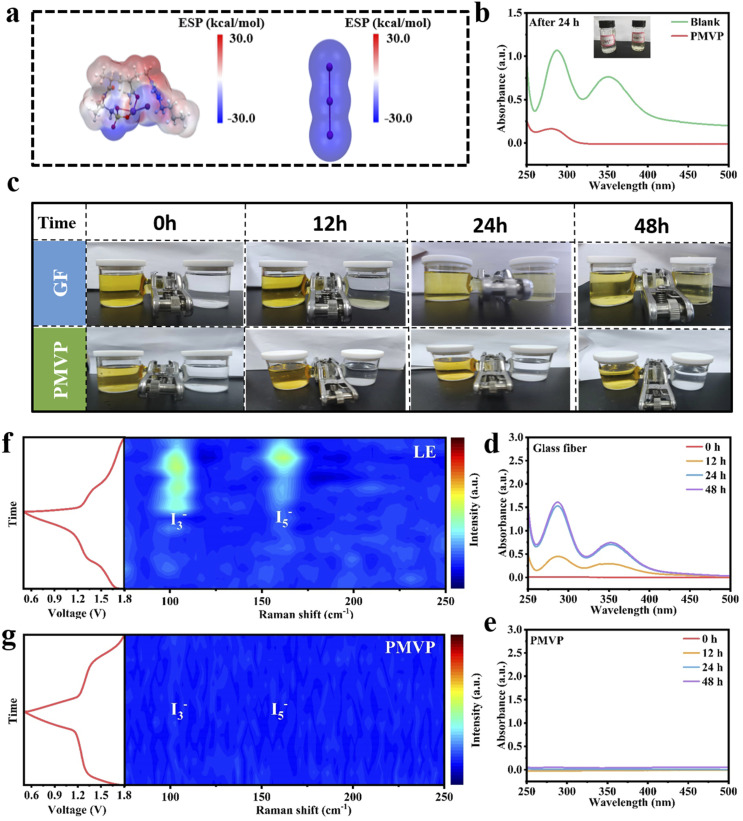
(a) ESP of PMVP and I_3_^−^. (b) UV-vis absorption spectra of polyiodide solutions. (c) Optical images of polyiodide shuttling. (d and e) UV-vis absorption spectra of the solution in the right chamber for (d) GF and (e) PMVP separators. (f and g) *In situ* Raman spectra in (f) LE and (g) PMVP electrolyte.

As for the cathode, the intrinsically low conductivity and insufficient structural stability of iodine tended to generate soluble polyiodide species. The uncontrolled diffusion of these intermediates between electrodes largely deteriorated the electrochemical performance and reversible capacity of zinc–iodine batteries.^42^ To explore the underlying mechanism that PMVP suppressed the shuttle effect derived from polyiodide species, theoretical calculations were performed to obtain the electrostatic potential (ESP) of PMVP, as well as the ESP of I_3_^−^ and PAM (Fig. 6a). The calculation results showed that the electrostatic potential of PMVP underwent a significant change compared to PAM (Fig. S22), which arose from the –SO_3_^−^ and imidazole moieties in the PMVP electrolyte. The –SO_3_^−^ functional groups in PMVP carried a negative potential, which could exert electrostatic repulsion on I_3_^−^ polyiodides. The PMVP electrolyte effectively repelled polyiodide intermediates away from the electrode surface *via* electrostatic repulsion, and their activity space was strictly confined to the cathode region. In contrast, the ESP of the imidazole groups in PMVP was opposite to that of I_3_^−^, suggesting that imidazole groups could adsorb polyiodides through electrostatic attraction. Through this adsorption, polyiodides were firmly locked. The synergistic effect of these anionic and cationic groups significantly inhibited the shuttle reaction of polyiodides—this mechanism was consistent with the synergistic coordination effect regulating zinc deposition analyzed earlier.

To further verify the polyiodide adsorption performance of PMVP hydrogel, the polyiodide solution was prepared by dissolving KI and I_2_ in deionized water at a molar ratio of 1 : 1. PMVP hydrogel was immersed in 3 mL polyiodide solution (2 mM). After 24 h of adsorption, UV-vis absorption spectroscopy was performed on the blank polyiodide solution and the polyiodide solution soaked with PMVP. UV-vis spectra demonstrated that the absorption peak of polyiodides in the PMVP system was significantly suppressed (Fig. 6b), confirming that PMVP exhibited a remarkable adsorption effect on polyiodides through its functional groups. This effectively prevented polyiodides from shuttling through the electrolyte to the zinc anode and avoided adverse reactions between polyiodides and zinc. Moreover, an H-type electrolytic cell was employed to simulate the shuttle behavior of polyiodides in zinc–iodine batteries.^41^ Specifically, 5 mM polyiodide solution (prepared with the same KI/I_2_ molar ratio of 1 : 1) and deionized water were added to the left and right chambers of the H-type cell, respectively, and the two chambers were separated by GF and PMVP (Fig. 6c). UV-vis tests were conducted to detect the polyiodide absorption signals at the initial state, 12 h, 24 h, and 48 h. Rapid shuttling of polyiodides from the left side to the right solution was observed with the GF separator, as reflected by the obvious absorption signal in the right chamber (Fig. 6d). In contrast, under the action of the PMVP hydrogel, no significant polyiodide absorption signal was detected in the right chamber (Fig. 6e), indicating that polyiodides on the left side were completely blocked at the hydrogel interface and could not shuttle to the right solution. In addition, UV-vis tests were performed on the three control hydrogels (Fig. S23). The results indicated that neutral PAM restricted polyiodide permeation solely through a simple physical screening effect, and significant polyiodide leakage was observed after 48 hours. The anionic PMP, containing only sulfate groups, blocked polyiodide through electrostatic repulsion, whereas the cationic PMV, based on imidazole, captured polyiodide through electrostatic adsorption; consequently, both PMP and PMV exhibited moderate barrier efficiency, further confirming the barrier effect of PMVP on polyiodides. To evaluate the effectiveness of PMVP hydrogel in inhibiting polyiodide shuttling in practical battery systems, Zn//I_2_ full cells based on the AC@I_2_ cathode were constructed. *In situ* Raman spectroscopy was utilized to track polyiodide species at the zinc anode–electrolyte interface, to compare the performance of LE and PMVP electrolyte in suppressing polyiodide shuttling. For the Zn//I_2_ full cell with LE, I_3_^−^ and I_5_^−^ were clearly identified during the charge–discharge process (Fig. 6f), indicating severe polyiodide shuttling. In comparison, no significant polyiodide signals were found in the cell with PMVP electrolyte (Fig. 6g), demonstrating that PMVP had a strong constraint on polyiodides and could substantially inhibit their shuttle behavior. Both electrostatic potential calculations and the above experiments confirmed that the anionic and cationic functional groups of PMVP can trap polyiodides at the cathode interface through electrostatic attraction and electrostatic repulsion, preventing them from shuttling to the zinc anode through the electrolyte. This effect was combined with the optimized zinc deposition behavior, interface stability, and cycle performance of PMVP electrolyte, enabling stable capacity retention in the Zn//PMVP//I_2_ full cell. These results further verified that PMVP electrolyte can comprehensively solve the key bottlenecks of zinc–iodine batteries, offering significant promise for practical applications.

**Fig. 7:**
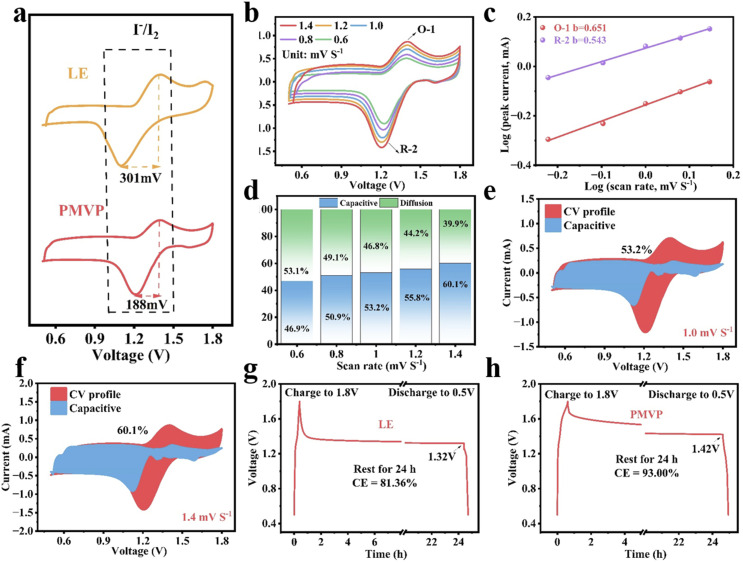
(a) CV curves of Zn//LE//I_2_ and Zn//PMVP//I_2_ batteries. (b) CV curves of the Zn//PMVP//I_2_ battery at different scan rates. (c) Calculated *b*-values. (d) Diffusion and capacitive contributions in PMVP electrolyte at different scan rates. (e and f) Schematic diagrams of capacitive contribution of PMVP electrolyte. (g and h) Self-discharge curves of Zn//LE//I_2_ and Zn//PMVP//I_2_ batteries.

To better understand the reaction kinetics at the electrode interface, CV was conducted for Zn//LE//I_2_ and Zn//PMVP//I_2_ batteries. At 1.0 mV s^−1^, the CV curves of Zn//LE//I_2_ and Zn//PMVP//I_2_ (Fig. 7a) showed one redox pair corresponding to the I^−^/I_2_ process.^43^ In comparison of the I^−^/I_2_ redox peaks, the PMVP electrolyte exhibited a smaller peak potential shift, demonstrating that the incorporation of PMVP lowered the electrode polarization of the zinc–iodine battery. CV measurements were recorded for the Zn//PMVP//I_2_ battery at 0.6 to 1.4 mV s^−1^ (Fig. 7b). The redox peaks remained well defined with negligible variation, demonstrating fast reaction kinetics in the Zn//PMVP//I_2_ battery. To further explore the iodine conversion kinetics, the correlation between redox peaks and scan rates was investigated *via* the peak current density, based on the correlation between the peak current (*i*) and the scan rate (*ν*):

1 *i* = *av*^*b*^

where *a* and *b* are adjustable parameters. The *b*-value was determined from the slope of the log *i*–log *ν* plot. The *b*-values derived from O-1 and R-2 were 0.651 and 0.543, respectively (Fig. 7c), suggesting that the iodine electrode process was co-controlled by capacitive and diffusion processes. The relatively moderate *b*-values of O-1 and R-2 demonstrated the satisfactory redox kinetics for I^−^/I_2_ reactions.^44^ Additionally, calculations of capacitive and diffusion contributions were performed, which could be determined by the following equation:

2 *i* = *k*_1_*v* + *k*_2_*v*^1/2^

where *k*_1_*v* and *k*_2_*v*^1/2^ represent capacitive and diffusion contributions, respectively. Fig. 7d and S24 quantified the capacitive and diffusion contributions of the Zn//PMVP//I_2_ battery. The capacitive contribution increased from 46.9% to 60.1% within this scan rate range, confirming that the iodine electrode process was co-controlled by capacitive and diffusion processes. When the scan rate exceeded 0.8 mV s^−1^, the interfacial capacitive behavior became dominant, and a higher capacitive contribution led to superior high-rate performance, which was attributed to the adsorption and blocking of polyiodides by the anionic and cationic functional groups of PMVP and to the inhibition of iodine shuttling by the hydrogel (Fig. 7e and f). CE obtained from long-term standing tests acted as a critical index to estimate the polyiodide shuttle effect. The results indicated that the Zn//PMVP//I_2_ battery maintained a CE of 93.00% after 24 h of standing (Fig. 7g and h), which was superior to that of the Zn//LE//I_2_ battery (81.36%). This result reflected the excellent ability of PMVP electrolyte to inhibit polyiodide shuttling.

### 2.7. Zn//I_2_ full batteries and pouch batteries

**Fig. 8:**
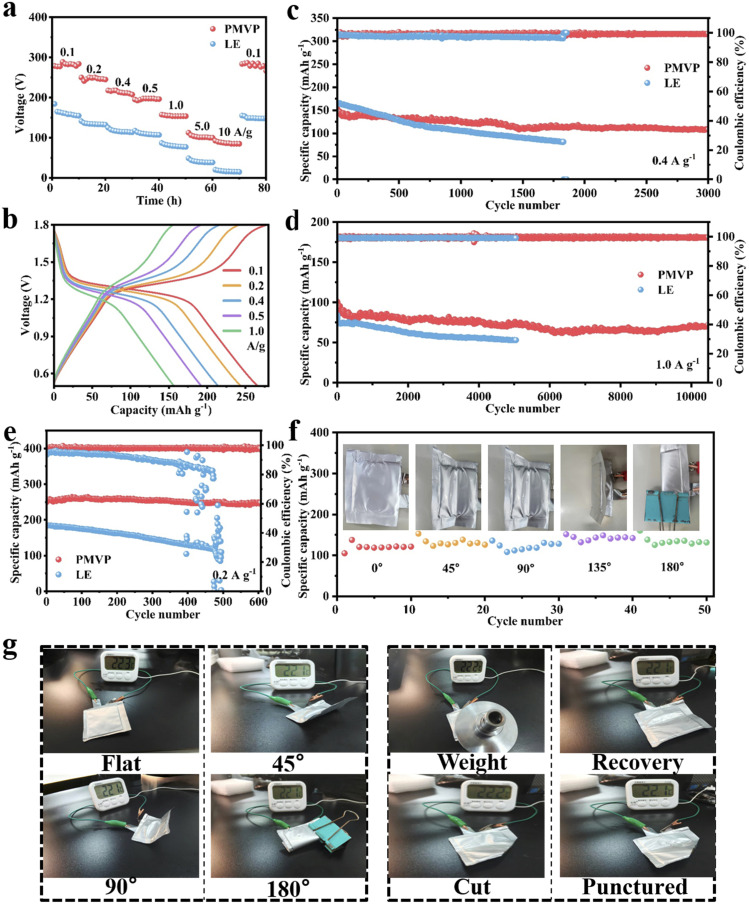
(a) Rate performance. (b) Voltage–capacity curves. (c–e) Cycle life at 0.4 A g^−1^, 1.0 A g^−1^ and 0.2 A g^−1^. (f) Capacity retention of Zn//I_2_ pouch batteries. (g) Zn//I_2_ pouch batteries powering a thermometer under different conditions.

The stability and cyclability of Zn//PMVP//I_2_ batteries were systematically examined to evaluate their overall performance. First, the rate performance of the Zn//PMVP//I_2_ battery was assessed within 0.5–1.8 V. Fig. 8a reveals that the capacity of Zn//PMVP//I_2_ batteries was superior to that of LE-based batteries at all tested current densities. Moreover, after undergoing a series of current density variations, the capacity of Zn//PMVP//I_2_ batteries almost recovered to the initial capacity when the current density was restored to 0.1 A g^−1^, demonstrating the superior rate performance of PMVP hydrogel electrolyte. The voltage–capacity curves at different current densities (Fig. 8b and S25) demonstrated that Zn//PMVP//I_2_ batteries exhibited higher charge–discharge capacities with changes in current density. This further validated that the PMVP hydrogel suppressed polyiodide shuttling at different current densities. As shown in Fig. 8c, the Zn//PMVP//I_2_ full battery maintained a specific capacity of 108.31 mA h g^−1^ after 3000 cycles at 0.4 A g^−1^, with a capacity retention of 70.64% and an average CE of 99.20%. In comparison, the battery with LE decayed rapidly within 1800 cycles, with a capacity retention below 58.51%. At 1.0 A g^−1^, the Zn//PMVP//I_2_ battery still retained a specific capacity of 72.30 mA h g^−1^ after 10 000 cycles, achieving a capacity retention of 71.48% and an average CE of 99.50%. However, the LE-based battery showed fast capacity decay, and its capacity retention rate was less than 63.03% after 5000 cycles (Fig. 8d). This result further verified the excellent durability of Zn//PMVP//I_2_ batteries. Notably, at 0.2 A g^−1^, the Zn//PMVP//I_2_ battery delivered an initial high capacity of 250.50 mA h g^−1^ and remained stable for 500 cycles with a capacity retention rate as high as 98.97% (Fig. 8e), highlighting its excellent current stability.

To highlight the wide applicability of PMVP electrolyte, a Zn//PMVP//I_2_ flexible pouch battery was assembled. The Zn//PMVP//I_2_ pouch battery possessed good flexibility. Cycle tests were conducted at different bending angles and even at the 180° fully overlapped state. As displayed in Fig. 8f, the Zn//PMVP//I_2_ pouch battery maintained stable cycling, confirming its reliable flexible performance. To simulate practical application scenarios, the pouch battery was used to power a thermometer at different bending angles (Fig. 8g). It also maintained stable power supply for the thermometer under harsh conditions including heavy compression, puncture, and cutting. These results illustrated the promising application prospects of Zn//PMVP//I_2_ flexible batteries in wearable, portable, and harsh-environment energy storage devices. Additionally, the electrochemical performance of our Zn//I_2_ battery is systematically compared with previously reported aqueous zinc–iodine systems (Fig. S26 and Table S4), further demonstrating the superiority of the PMVP hydrogel electrolyte and its promising application potential.

## 3. Conclusions

In summary, we fabricated a novel polyzwitterionic hydrogel electrolyte with imidazolium cation and sulfonate anion moieties to successfully address the two key challenges of aqueous zinc–iodine batteries, including zinc dendrite formation and polyiodide shuttling. The polyzwitterionic hydrogel electrolyte exerted synergistic effects on the zinc anode and iodine cathode, thereby optimizing the battery performance. On the zinc anode, the –SO_3_^−^ groups electrostatically attracted Zn^2+^ and reshaped the solvation structure, while the imidazolium groups electrostatically repelled Zn^2+^, promoting the uniform deposition of Zn^2+^. This synergistic effect between anionic and cationic groups inhibited zinc dendrite growth. On the iodine cathode, the –SO_3_^−^ groups generated strong electrostatic repulsion on polyiodides, confining them to the cathode interface to prevent their shuttling through the electrolyte. Additionally, the imidazolium groups electrostatically adsorbed polyiodides, firmly locking them at the iodine cathode and further suppressing the polyiodide shuttle effect. The Zn//PMVP//Zn symmetric battery delivered a long-term cycling stability over 4500 h at 1 mA cm^−2^/1 mA h cm^−2^, 1000 h at 10 mA cm^−2^/1 mA h cm^−2^ and 450 h at 20 mA cm^−2^/1 mA h cm^−2^, demonstrating its effective zinc dendrite inhibition across different current densities. The Zn//PMVP//I_2_ full battery ran stably beyond 10 000 cycles at 1 A g^−1^ with a capacity retention of 71.48%. Furthermore, the Zn//PMVP//I_2_ flexible pouch battery operated stably under various extreme conditions and can continuously power a thermometer, laying a foundation for its practical application in flexible energy storage devices. This study offers an available strategy for the development of advanced hydrogel electrolytes to regulate dendrite growth and suppress polyiodide shuttle, enabling long-life zinc–iodine batteries.

## Author contributions

Bokang Yuan: conceptualization, investigation, writing – original draft, supervision. Fangfei Liu: conceptualization, data curation, funding acquisition, formal analysis, supervision, writing – review and editing. Yufan Lei: investigation. Minghui Xu: investigation. Yuting Xie: investigation. Huaren Fan: investigation. Feng Zeng: supervision. Xiong Liu: conceptualization, data curation, funding acquisition, formal analysis, supervision, writing – review and editing.

## Conflicts of interest

The authors declare no conflict of interest.

## Supplementary Material

SC-OLF-D6SC03860C-s001

## Data Availability

The data that support the findings of this study are available on request from the corresponding author, upon reasonable request. Supplementary information (SI) is available. See DOI: https://doi.org/10.1039/d6sc03860c.
