# Supplementary material for: Polyzwitterionic hydrogel electrolytes based on imidazolium cations and sulfonate anions enable stable Zn–I2 batteries by regulating Zn deposition and inhibiting polyiodide shuttle
Source: Chem Sci. 2026 Jun 26;17(33):15825–38. doi: 10.1039/d6sc03860c (PMC13344477; doi:10.1039/d6sc03860c)
Supplement: Supplementary file 1 [file SC-017-D6SC03860C-s001.pdf]

Supplementary Information (SI) for Chemical Science.
This journal is © The Royal Society of Chemistry 2026

Supporting Information

Polyzwitterionic hydrogel electrolytes based on imidazolium cation and sulfonate anion enable stable Zn–I_2 batteries by regulating Zn deposition and inhibiting polyiodide shuttle

Bokang Yuan, Fangfei Liu*, Yufan Lei, Minghui Xu, Yuting Xie, Huaren Fan, Feng Zeng, Xiong Liu*

State Key Laboratory of Chemistry and Utilization of Carbon Based Energy Resources, College of Chemistry, Xinjiang University, Urumqi, 830017, Xinjiang, PR China;

*Corresponding authors.

E-mail: liufangfei1214@163.com (F.F. Liu); liuxiong@xju.edu.cn (X. Liu)

Experimental Section

Materials

Acrylamide (AM, 98%), sodium 2-acrylamido-2-methyl-1-propanesulfonate (NaAMPS, 50 wt% in H_2O, 98%), 1-butyl-3-vinylimidazolium bromide (VBIMBr, 98%), N, N'-methylenebisacrylamide (MBA, 98%), zinc trifluoromethanesulfonate ($Zn(OTf)_2$, 98%), and 2-hydroxy-4'-(2-hydroxyethoxy)-2-methylpropiophenone (Irgacure2959, 98%) were purchased from Energy Chemical, Adamas-beta® and Macklin.

Synthesis of hydrogel electrolytes

A one-pot synthesis method was used to prepare hydrogel electrolytes. Specifically, 0.3 g NaAMPS and 0.301 g VBIMBr at an equimolar ratio were first

dissolved in 10 mL of deionized water. After these components were completely dissolved, 2 g of AM and 1 M $Zn(OTF)_2$ were introduced into the resulting solution, and the solution was thoroughly agitated until all substances were fully dissolved. Subsequently, 15 mg of MBA and 40 mg of Irgacure 2959 were added to the homogeneous solution in turn. After 30 minutes of continuous stirring to disperse the crosslinking agent and photoinitiator uniformly, ultrasonic treatment was carried out to remove air bubbles from the solution, this step is essential to prevent defects in the final hydrogel. The bubble-free solution was poured into the gap between two glass plates separated by adhesive tapes. The assembled setup was then irradiated with UV light for 10 minutes to trigger cross-linking polymerization, thus obtaining the cross-linked hydrogel (denoted as PMVP). For comparison, three control hydrogels (PMV, PMP, and PAM) were prepared using the same method as PMVP hydrogel, except for the following differences: PMV without NaAMPS, PMP without VBIMBr, and PAM hydrogel without both NaAMPS and VBIMBr.

Synthesis of AC@I_2 and preparation of its corresponding electrode

Initially, 1.0 g of iodine was blended with activated carbon (AC) in a 1:1 mass ratio, followed by grinding the mixture for 20 minutes to achieve homogeneous dispersion. The ground powder was placed in a glass vessel and maintained at 60 °C for 6 hours. Subsequently, the sample was blended with acetylene black and CMC at a mass ratio of 7:2:1, and deionized water was introduced as a solvent to yield a uniform slurry. Finally, the well-dispersed slurry was cast onto a titanium foil substrate. The iodine loading of the as-prepared cathode was measured to be approximately 0.8–1.5

mg cm^{-2}.

Mechanical Tests

Mechanical characterizations of the hydrogel electrolytes were carried out on a universal testing machine (ZQ-990LB). For tensile tests, the hydrogel samples were cut into dumbbell-shaped strips with dimensions of 25 mm (length) × 4 mm (width) × 2 mm (thickness), and the tensile rate was set at 50 mm·min^{-1}. For compression tests, cylindrical hydrogel samples with a diameter of 13 mm and a height of 7 mm were used, and the compression rate was fixed at 50 mm·min^{-1}.

The tensile stress (δ) of the samples was calculated using the following equation:

$$\delta = \frac{F}{A} \#(1)$$

where F is the tensile force (N), and A is the cross-sectional area of the sample (m^2).

The tensile strain (ε) was determined according to Equation (2):

$$\varepsilon = \frac{L - L_0}{L_0} \#(2)$$

where L is the length of the sample after stretching (m), and L_0 is the original length of the sample (m).

The adhesive strength of the hydrogels was measured via the lap shear test. The hydrogel samples were cut into cuboids with dimensions of 30 mm (length) × 15 mm (width) × 2 mm (thickness) and then sandwiched between two substrate materials. The test was conducted at a loading rate of 50 mm·min^{-1}, and the adhesive strength (P) was calculated using Equation (3):

$$P = \frac{F_{max}}{S}\#(3)$$

where F_{max} is the maximum load force (N), and S is the adhesion area (m^2).

Electrochemical Tests

Electrochemical evaluations were performed using CR2032 coin cells. In these cells, the hydrogel acted as both the electrolyte and the separator. For the liquid electrolyte cell ($Zn(OTF)_2$), an additional GF/D glass fiber separator (Whatman) was employed. The cycling performance of all cells was evaluated via galvanostatic charge-discharge (GCD) cycling and rate capability tests on a NEWARE CT-4008Tn battery testing system under different current and capacity densities to assess their electrochemical performance and long-term reliability.

Besides, Zn//Cu half-cells were assembled to conduct Zn plating/stripping experiments. Tafel tests were carried out on Zn//Zn symmetric cells at a scan rate of 5 $mV \cdot s^{-1}$ within the potential range of -0.1 V to 0.1 V. Cyclic voltammetry (CV) tests were performed on Zn//Cu half-cells at a scan rate of 5 mV s^{-1}, while linear sweep voltammetry (LSV) tests were conducted on Zn//stainless steel (SS) cells at the same scan rate.

Electrochemical impedance spectroscopy (EIS) measurements were performed at room temperature, with the hydrogel electrolyte sandwiched between two stainless steel electrodes. The ionic conductivity (σ) of the hydrogel electrolyte was calculated using Equation (1):

$$\sigma = \frac{L}{R \cdot S}\#(1)$$

where L is the thickness of the separator or hydrogel electrolyte (m), R is the ohmic

resistance (Ω), and S is the contact area between the electrolyte and the electrode (m^2). The zinc-ion transference number ($t_{Zn^{2+}}$) was measured by applying a step voltage of 10 mV for 3000 s combined with EIS tests, and calculated according to Equation (2):

$$t_{Zn^{2+}} = \frac{I_S(\Delta V - I_0 R_0)}{I_0(\Delta V - I_S R_S)} \#(2)$$

where ΔV is the applied step voltage, I_0 and I_S are the initial current and steady-state current, respectively, and R_0 and R_S are the impedance values before and after applying the step voltage, respectively.

The activation energy (Ea) was determined using EIS data obtained at different temperatures and the following equation:

$$\frac{1}{R_{ct}} = Aexp\left(-\frac{E_a}{RT}\right) \#(3)$$

where *Rct* is the charge transfer resistance, A is a pre-exponential factor, R is the gas constant, and T is the absolute temperature in Kelvin.

Characterization

The chemical valence, elemental composition, and micro-morphology of PMVP were determined by means of X-ray photoelectron spectroscopy (XPS, Thermo Fisher Scientific ESCALAB 250Xi), Fourier transform infrared (FTIR) spectroscopy (Bruker VERTEX70), and scanning electron microscopy (SEM, HITACHI SU8010), respectively. For the zinc anode, its surface microstructural features were probed through a combination of atomic force microscopy (AFM, Bruker Dimension ICON) and X-ray powder diffraction (Bruker D8-Advance). Tensile mechanical characterization was implemented on strip-shaped hydrogel electrolyte specimens (25

mm in length, 4 mm in width, and 2 mm in thickness) using a ZQ-990LB universal testing machine, with the tensile rate fixed at 50 mm·min^{-1}. Whereas the adhesive capability of the hydrogel was evaluated via the lap shear test protocol.

Theoretical computational details

Molecular dynamics simulations (MD): To investigate the solvation dynamics of Zn^{2+} ions, MD simulations were performed for four distinct electrolyte systems: a standard aqueous electrolyte (LE) containing only zinc trifluoromethanesulfonate ($Zn(OTF)_2$), and three polymer hydrogel electrolytes denoted as PMV (P(AM-VBIMBr)), PMP (P(AM-NaAMPS)), and PMVP (P(AM-VBIMBr-NaAMPS)). All simulations were conducted using the Gromacs program suite. A hybrid force field approach was adopted: the Merz ion force field, was used for Zn^{2+} cations, the OPC3 model for water molecules,[1] and the OPLS-AA framework for the trifluoromethanesulfonate (OTF^{-}) anion.[2] Force field parameters for the polymer chains, including acrylamide (AM), 1-butyl-3-vinylimidazolium bromide (VBIMBr), and sodium 2-acrylamido-2-methyl-1-propanesulfonate (NaAMPS), were generated using the mSeminario method with atomic charges derived from the RESP model. All molecular topologies were generated via the AuToFF web server.

Each electrolyte system contains 100 zinc cations, and the molar ratios of the components are consistent with the results observed in our experimental setup. The detailed number of each molecule and ion in the LE and PMVP MD systems are summarized in Table S1 and Table S2. Initial cubic simulation boxes (8×8×8 nm^3) were constructed using Packmol, populated with molecules at compositions matching the

experimental formulations. The degree of polymerization was set to 20. Each system underwent energy minimization, followed by a 1 ns annealing stage to gradually heat the system to 298.15 K. Subsequently, a 20 ns production run in the NPT ensemble was performed. Temperature was maintained at 298.15 K using the velocity-rescale thermostat (τ = 1 ps), and pressure was controlled at 1 bar using the Berendsen barostat. Periodic boundary conditions were applied, and long-range electrostatics were handled by the PME method with a 15 Å cutoff. The final 10 ns of the trajectory were used for analyzing the radial distribution function (RDF) and coordination numbers (CN).

Density functional theory (DFT): All quantum chemical calculations were performed to determine binding energies and desolvation energies using density functional theory (DFT). The geometries of all molecular clusters were optimized using the PBE0 hybrid functional and the def2-TZVP basis set. Empirical dispersion effects were included via the DFT-D3 correction.[3] The SMD implicit solvent model was employed to simulate an aqueous environment. All DFT calculations were carried out using the Gaussian 16 program suite.[4]

To quantitatively assess the interaction strength between Zn^{2+} ions and the various polymer components, we calculated the binding energies (E_bind). A more negative binding energy signifies a stronger, more thermodynamically favorable interaction[4]. The results are summarized in Table 1. The binding energy (E_bind) between a Zn^{2+} ion and a ligand (water or polymer segment) was calculated as:

$$E\_bind = E\_(Zn^{2+}\text{-ligand}) - E\_(Zn^{2+}) - E\_(ligand)$$

where E_(Zn^{2+}-ligand), E_(Zn^{2+}), and E_(ligand) are the total energies of the optimized complex, the isolated Zn^{2+} ion, and the isolated ligand, respectively.

COMSOL Multiphysics: The electrochemical tertiary current distribution model in COMSOL was employed for the numerical solution in this project. The tertiary current distribution model was solved on the basis of the Nernst-Planck equations.

$$\nabla \cdot \mathbf{i}_l = F\sum_i z_i R_i + Q_l$$
$$\mathbf{i}_l = -\delta_l \nabla \phi_l$$
$$\nabla \cdot \mathbf{i}_s = -F\sum_i z_i R_i + Q_s$$
$$\mathbf{i}_s = -\delta_s \nabla \phi_s$$
$$\frac{\partial c}{\partial t} + \nabla \cdot \mathbf{J} + \mathbf{u} \cdot \nabla c = R$$
$$\mathbf{J} = \sum_{i=1}^{N} \left(-D_i^m c_i + c_i z_i u_{m,i} F \nabla \phi\right)$$

In the equations: $\boldsymbol{is}$ and $\boldsymbol{il}$ represent the solid-phase electron current and the liquid-phase ion current, respectively; $\delta s$and δl denote the electrical conductivities of the solid and liquid phases, respectively; Qs and Ql are the charge quantities in the solid and liquid phases, respectively; ϕs and ϕl are the electric potentials in the solid and liquid phases, respectively. In the equations: the subscript i denotes the species number, c represents concentration, $\boldsymbol{J}$ is the flux of the species, $\boldsymbol{i}$ is the current density, R is the reaction source, which is set to 0 for this study. F is the Faraday constant, z is the charge number of the species, um is the mobility, which satisfies the Nernst-Einstein equation. V is the potential, and D is the diffusion coefficient of the species.

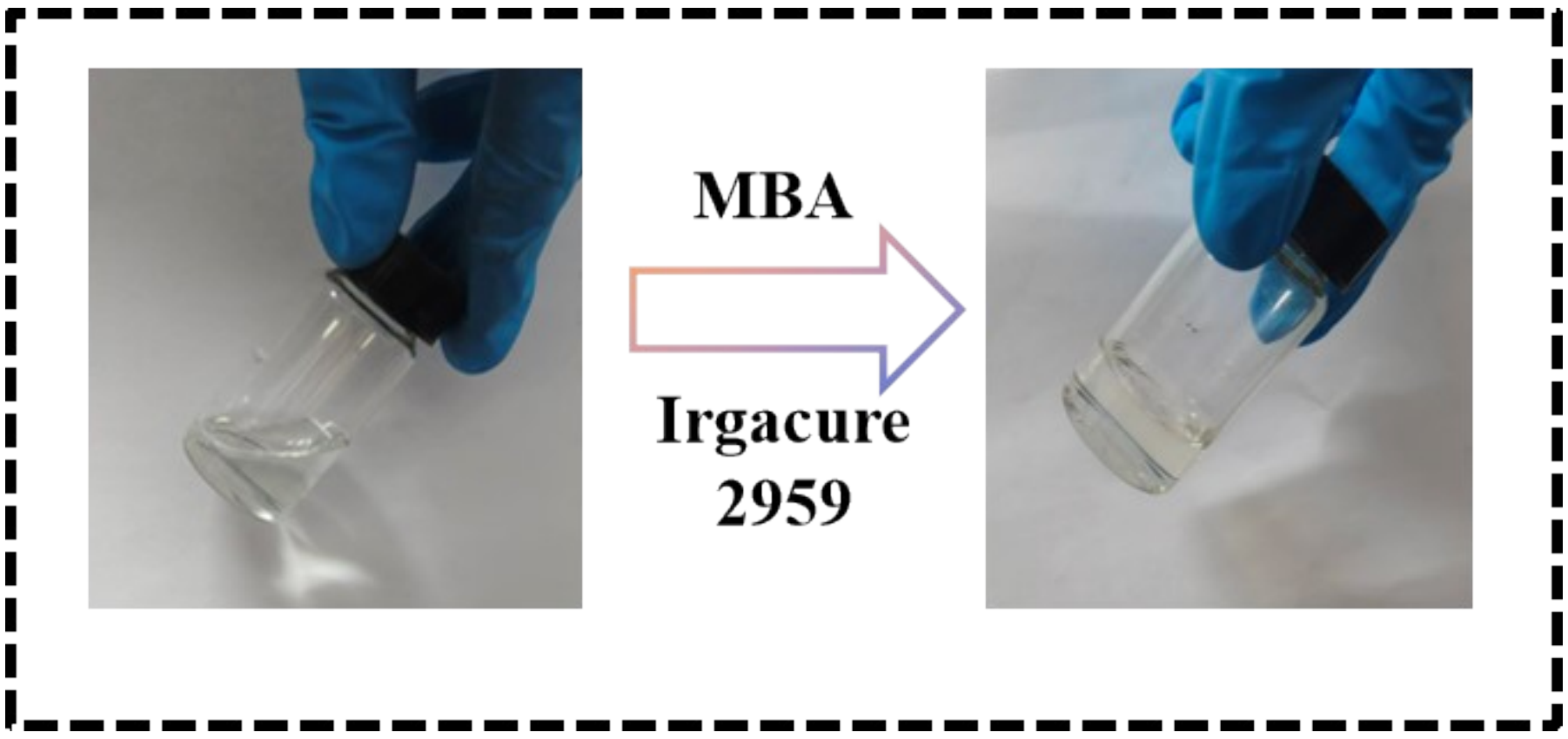

Fig. S1 Photograph of actual hydrogel preparation process.

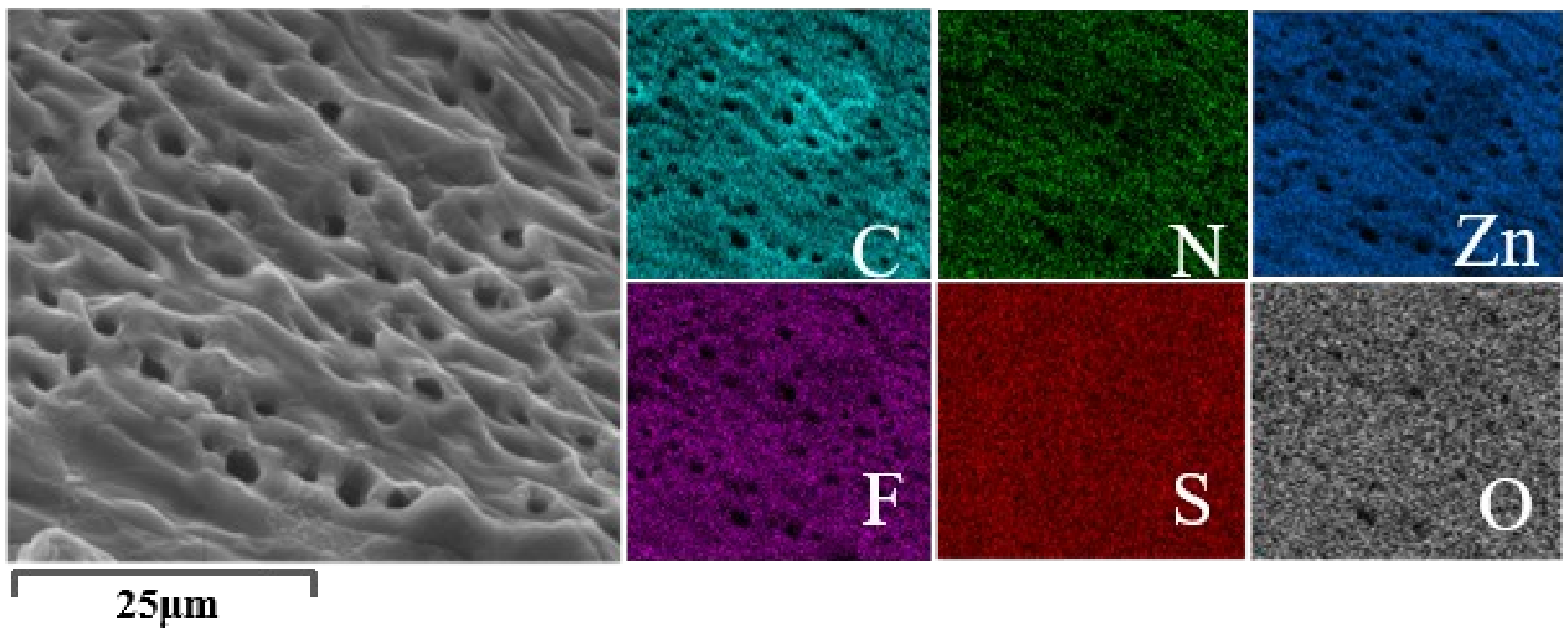

Fig. S2 SEM image of the hydrogel and elemental mappings of C, F, N, S, Zn, O.

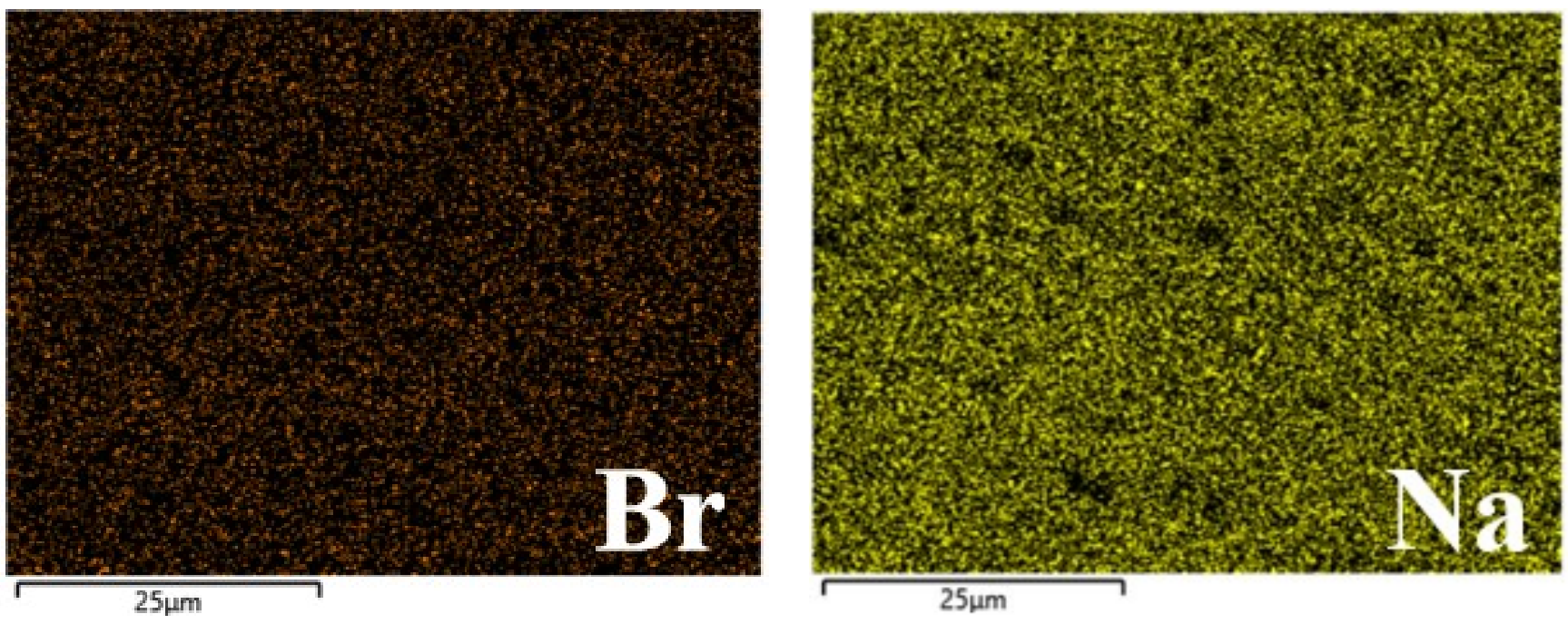

Fig. S3 EDS supplement for PMVP- hydrogel.

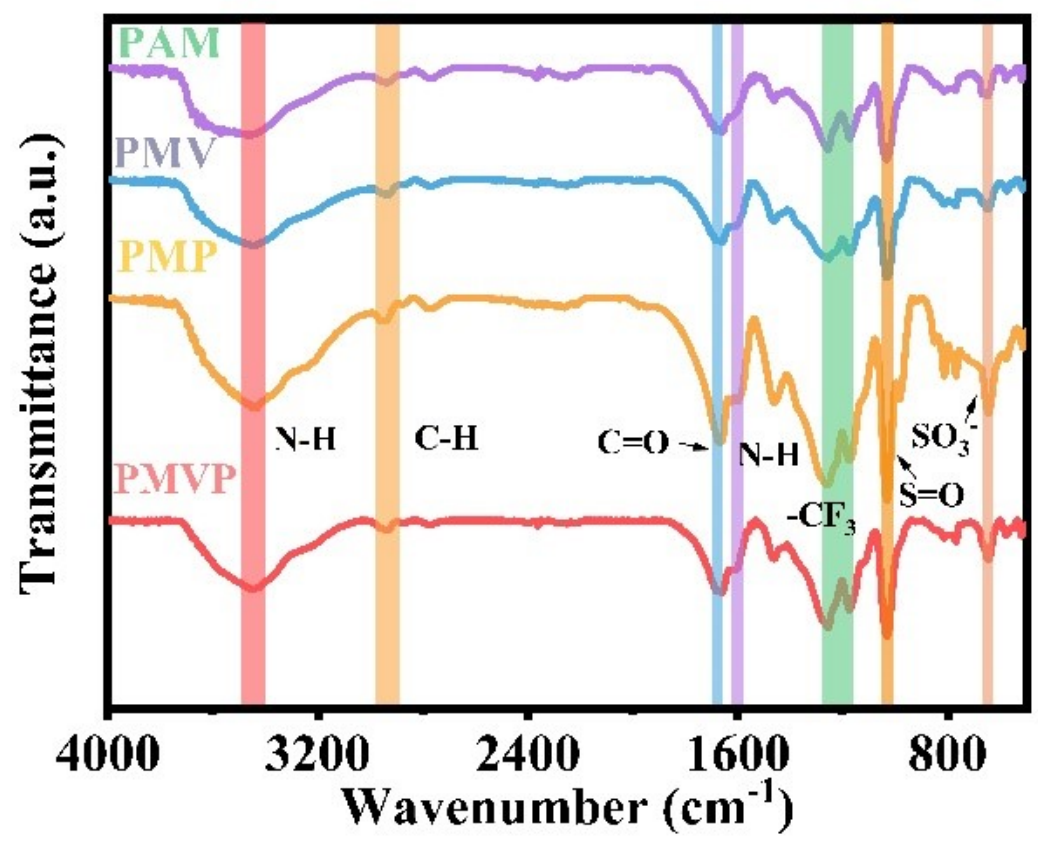

Fig. S4 FT-IR spectra of PMVP, PMP, PMV, PAM.

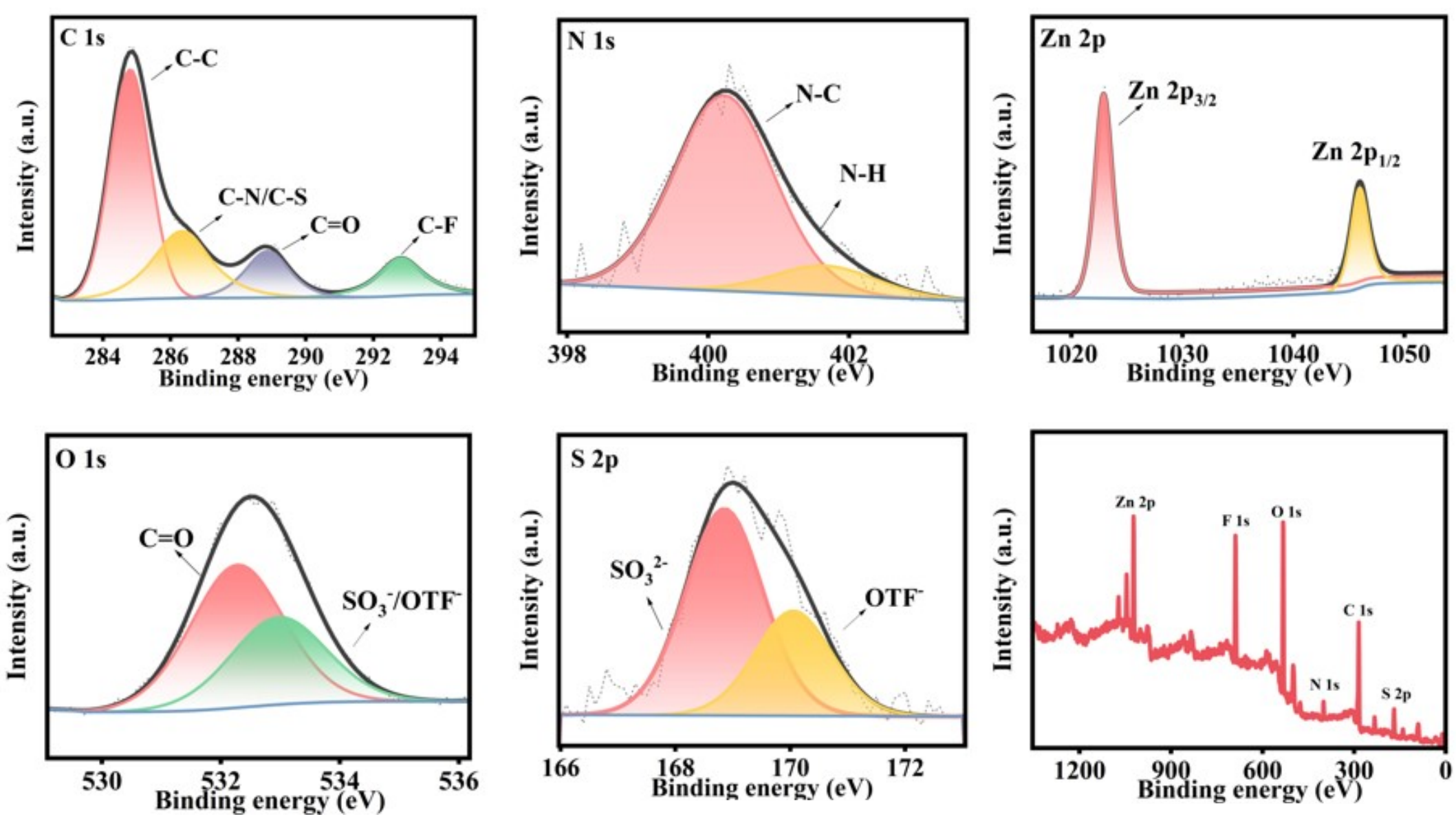

Fig. S5 XPS for PMVP- hydrogel.

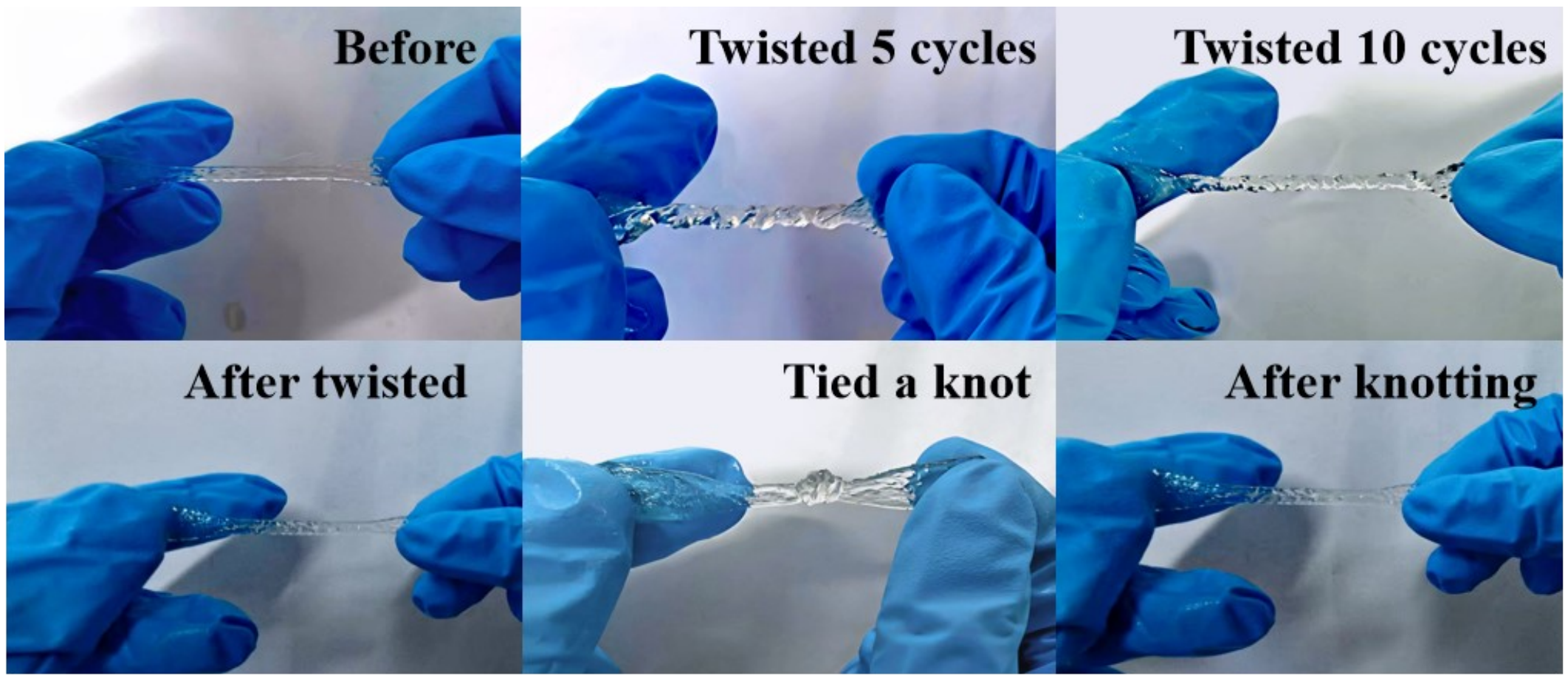

Fig. S6 Mechanical properties of PMVP- hydrogel in different conditions.

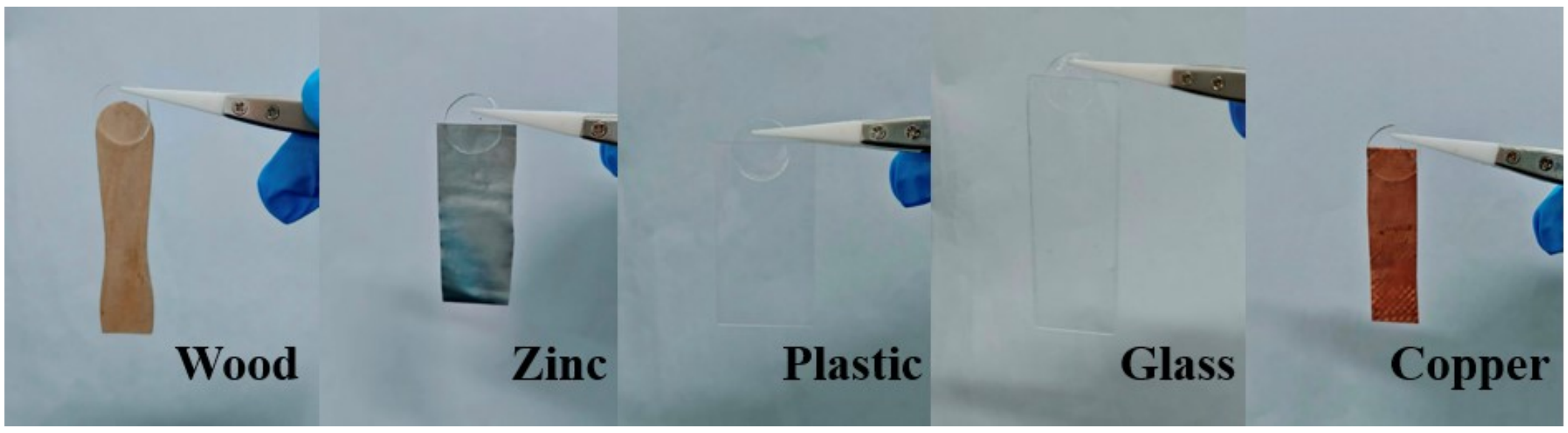

Fig. S7 Photograph of self-adhesive hydrogel adhered to the substrate surface.

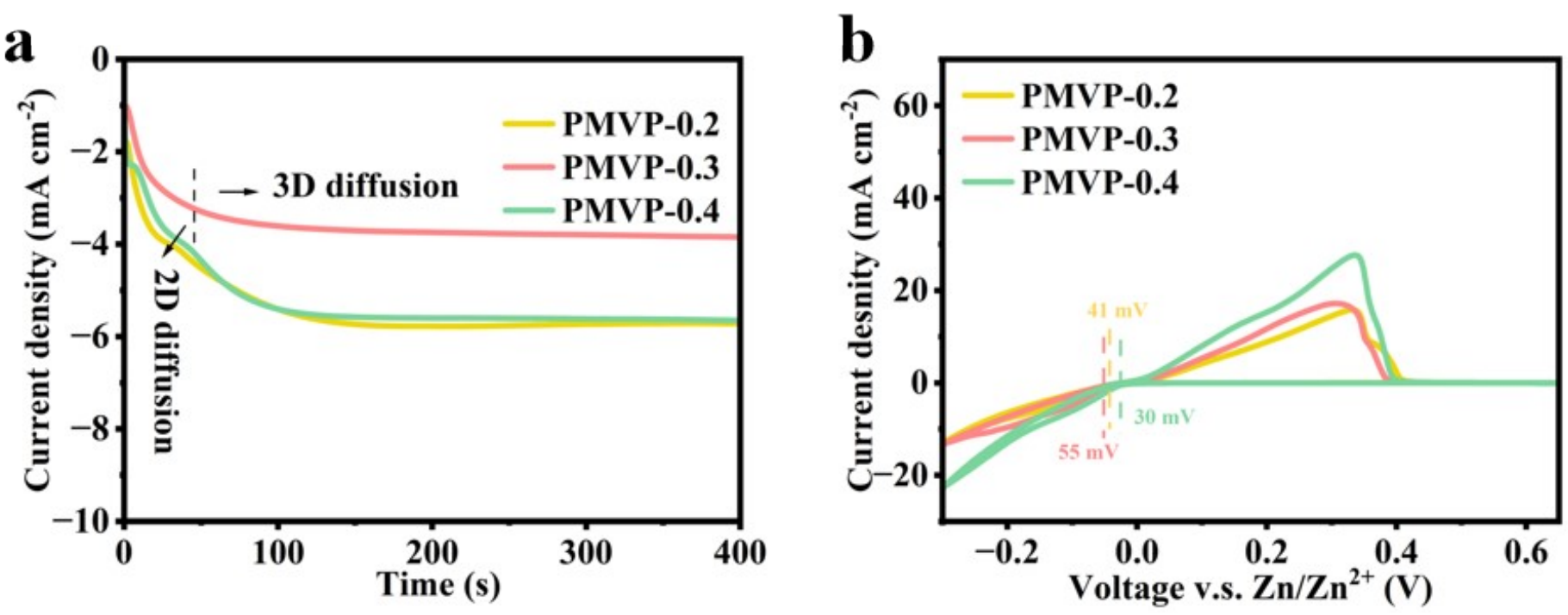

Fig. S8 (a) Tafel plots and (b) CV curves of PMVP at different dosages

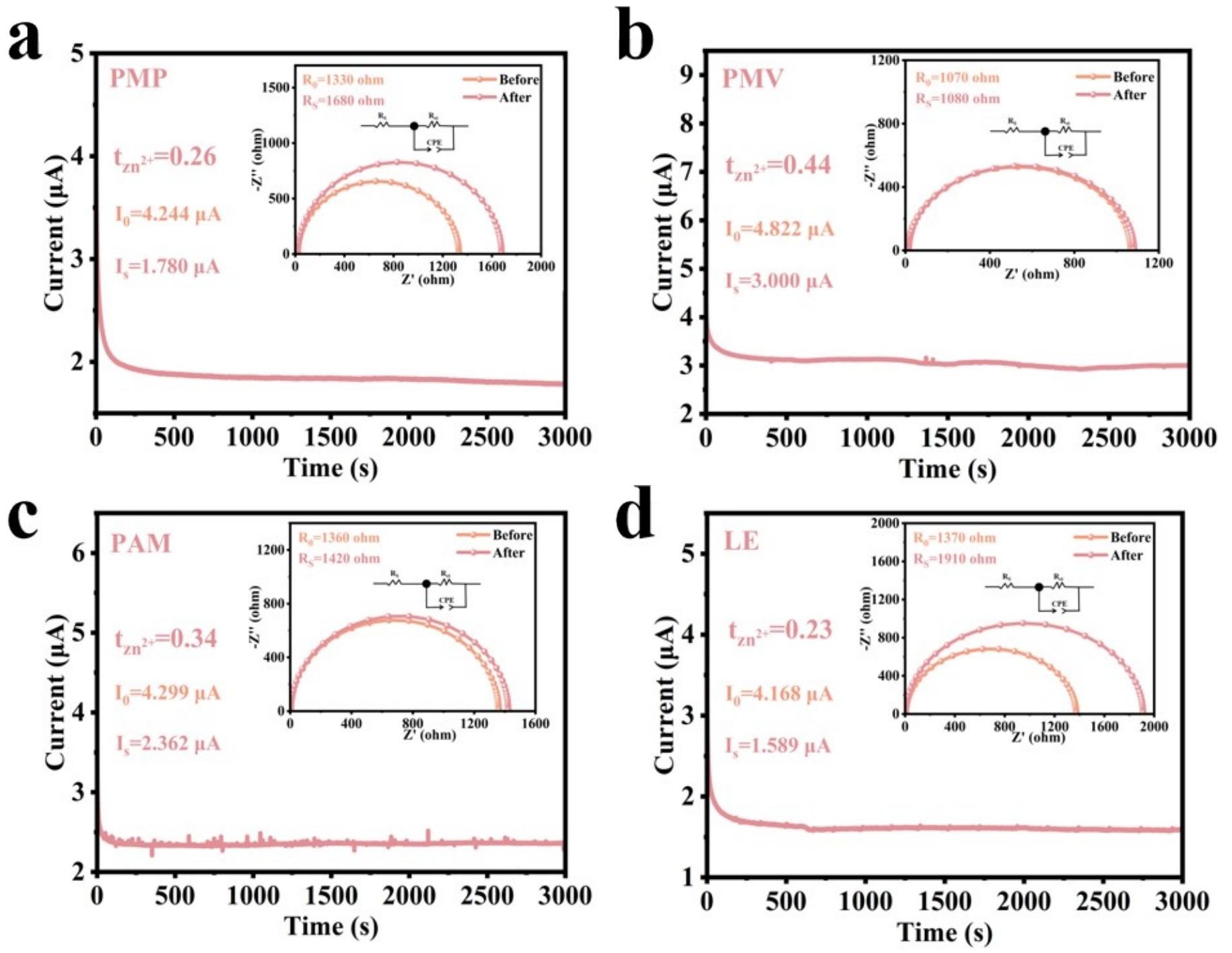

Fig. S9 I-t curves of symmetric cells with PMP (a), PMV (b), PAM (c), LE(D) electrolytes (inset: Nyquist plots before and after polarization).

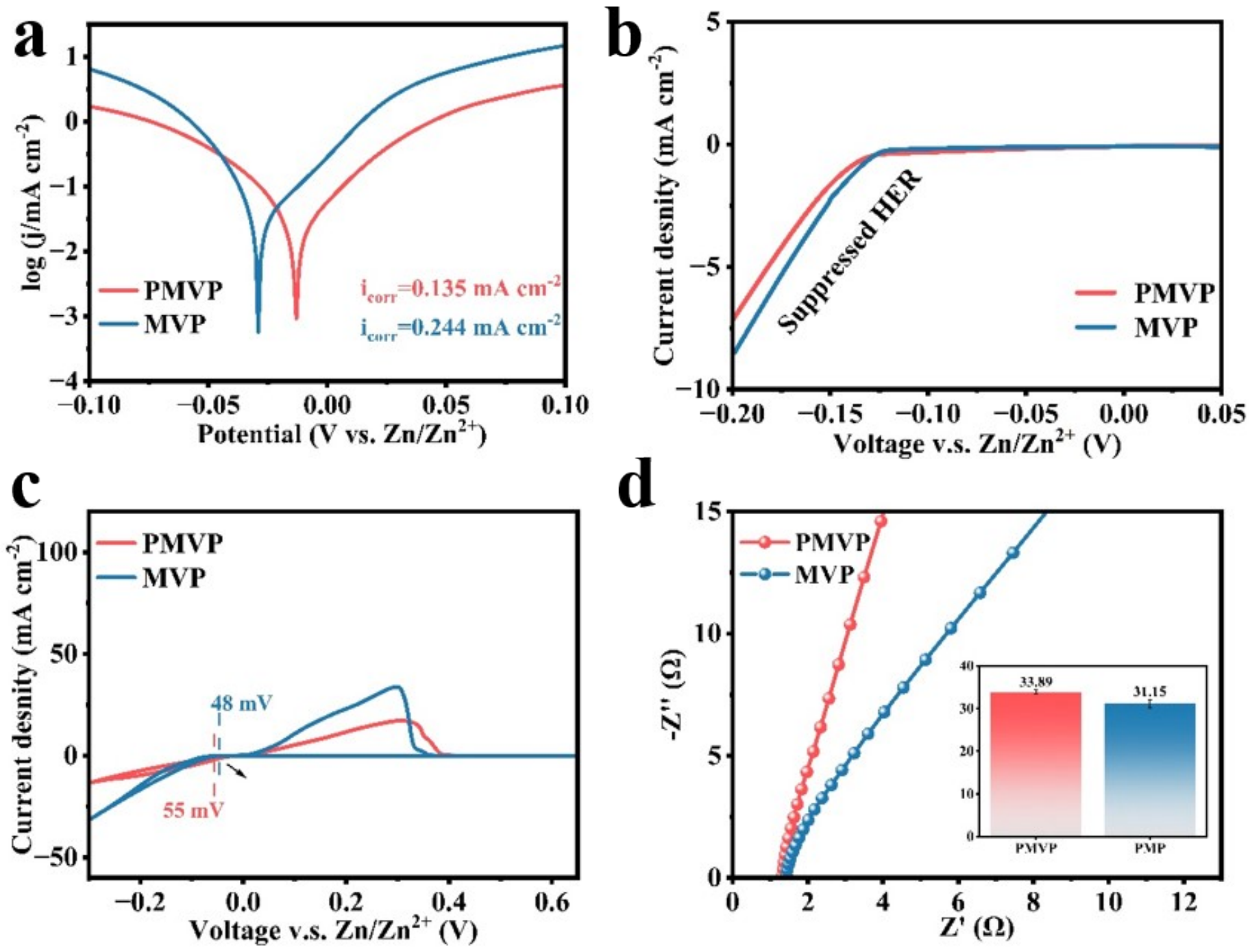

Fig. S10 (a) Tafel plots, (b) LSV curves, (c) CV profiles and (d) Nyquist plots of PMVP and MVP.

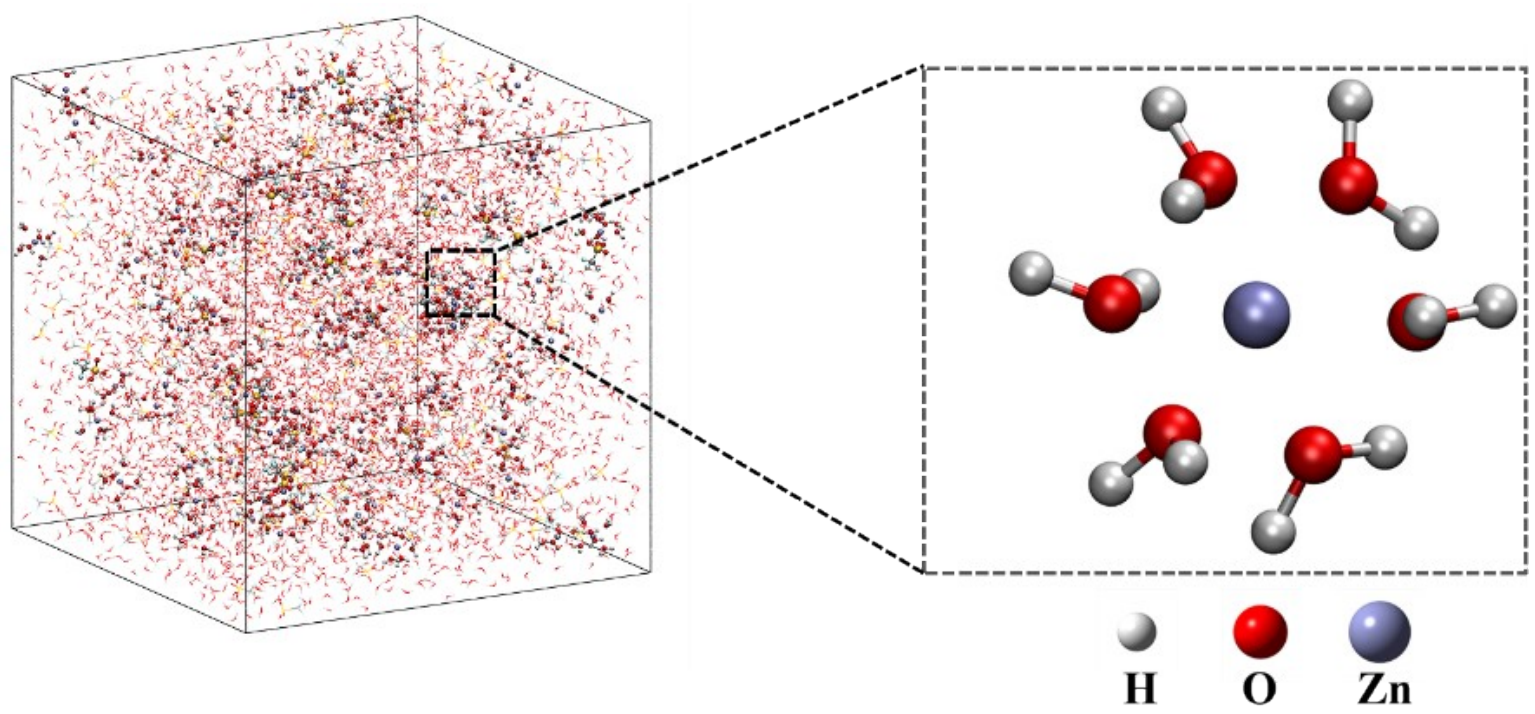

Fig. S11 MD simulation snapshot and representative solvation structure of Zn^{2+} in PMVP hydrogel electrolyte.

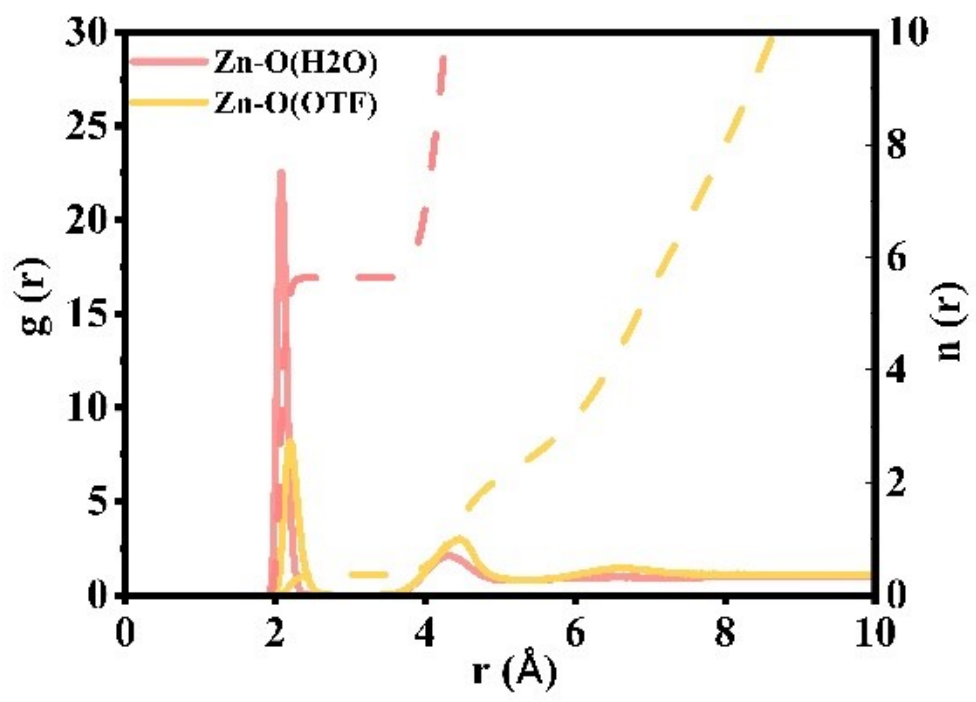

Fig. S12 RDF and coordination number of the LE hydrogel electrolyte.

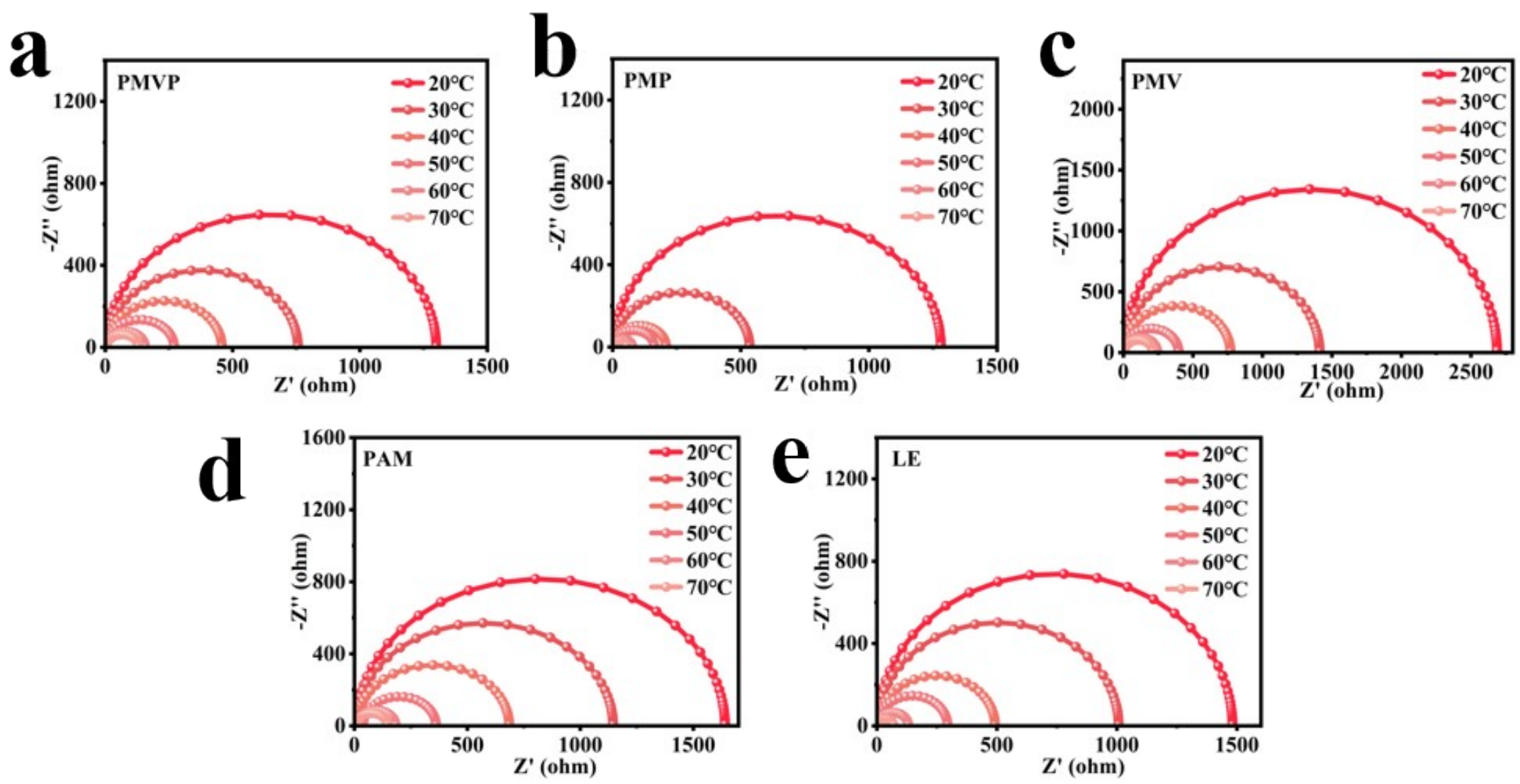

Fig. S13 Nyquist plots of (a) Zn//PMVP//Zn, (b) Zn//PMP//Zn, (c) Zn//PMV//Zn, (d) Zn//PAM//Zn, (e) Zn//LE//Zn at different temperatures.

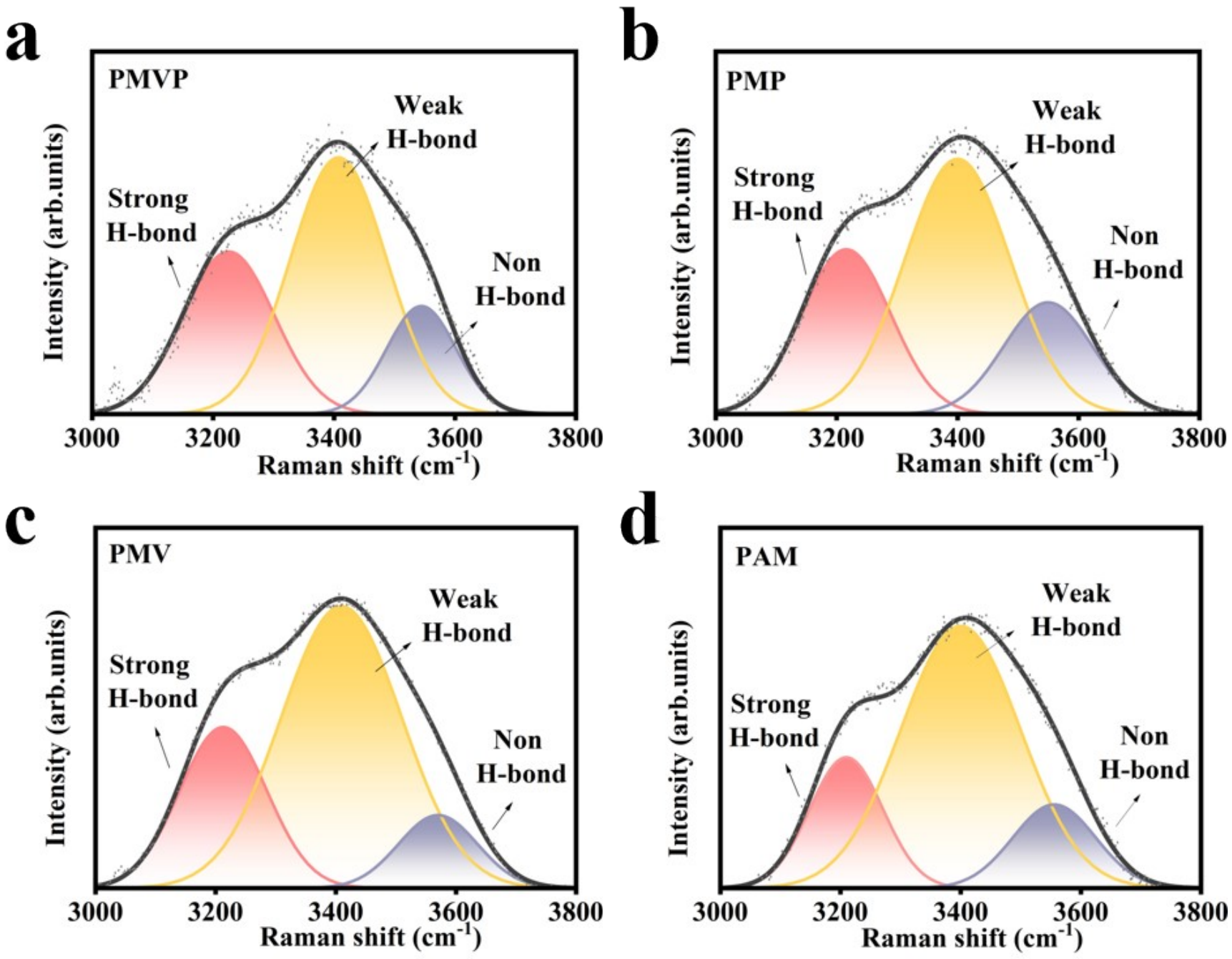

Fig. S14 Curve fitting results of the Raman spectra for (a) PMVP, (b) PMP , (c) PMV, and (d) PAM

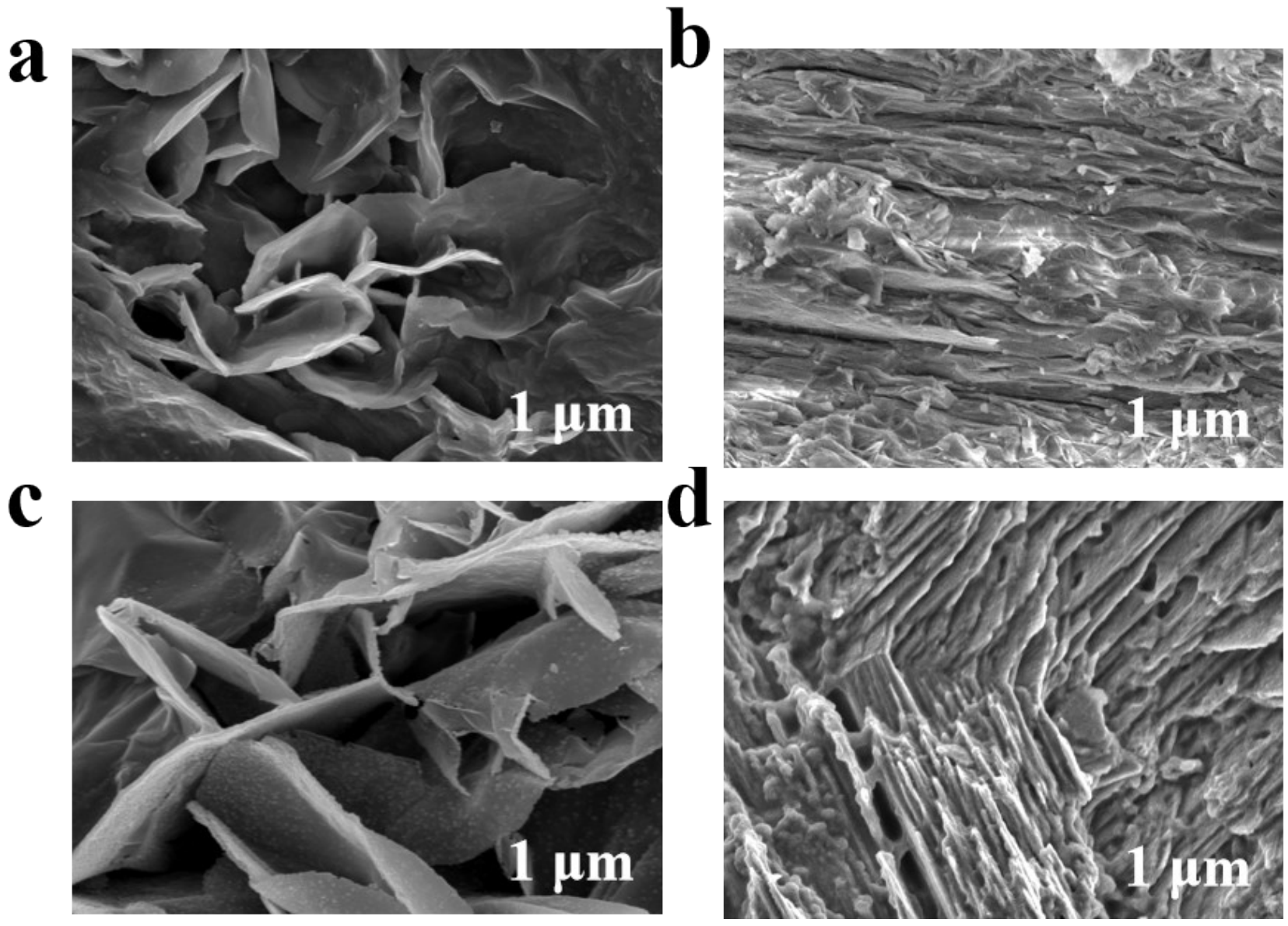

Fig. S15 SEM images of Zn anodes after 50 h of cycling with (a) LE and (b) PMVP

electrolytes and SEM images of Zn anodes after 200 h of cycling with (a) LE and (b) PMVP electrolytes at 1 mA cm^{-2} and 1 mAh cm^{-2} respectively.

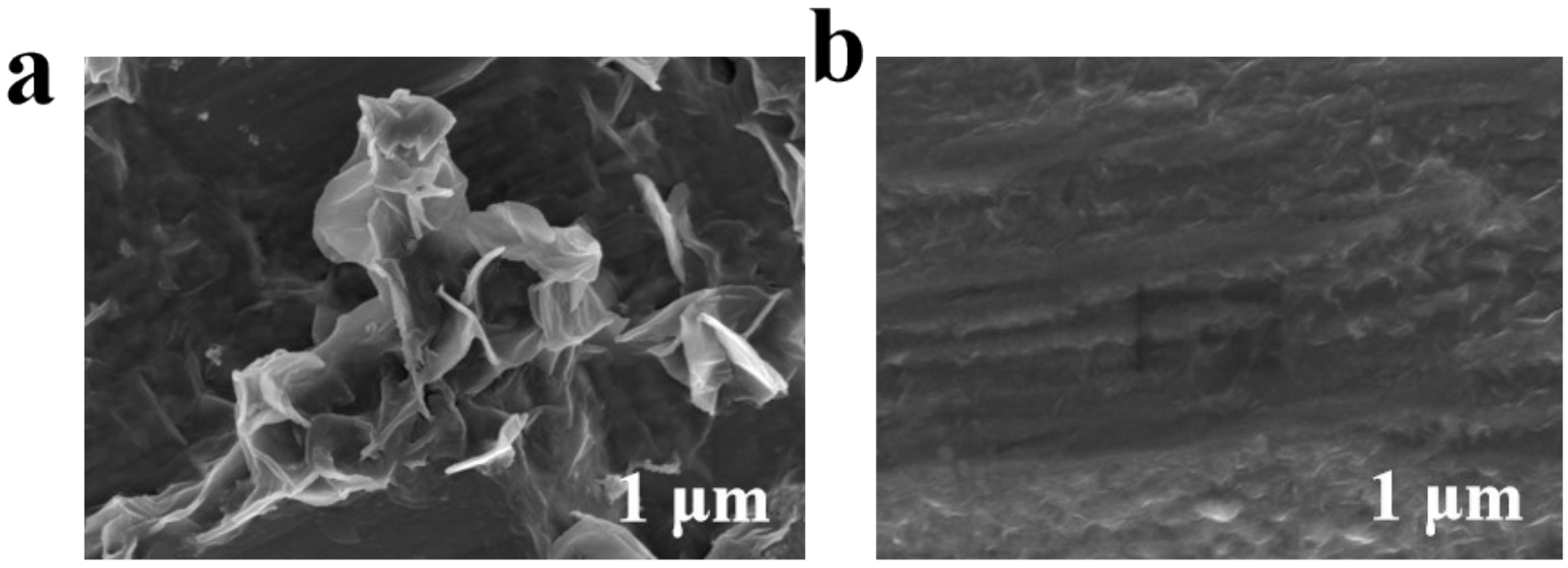

Fig. S16 SEM images of Zn anodes after 100 h of cycling with (a) LE and (b) PMVP electrolytes at 2 mA cm^{-2} and 2 mAh cm^{-2} respectively.

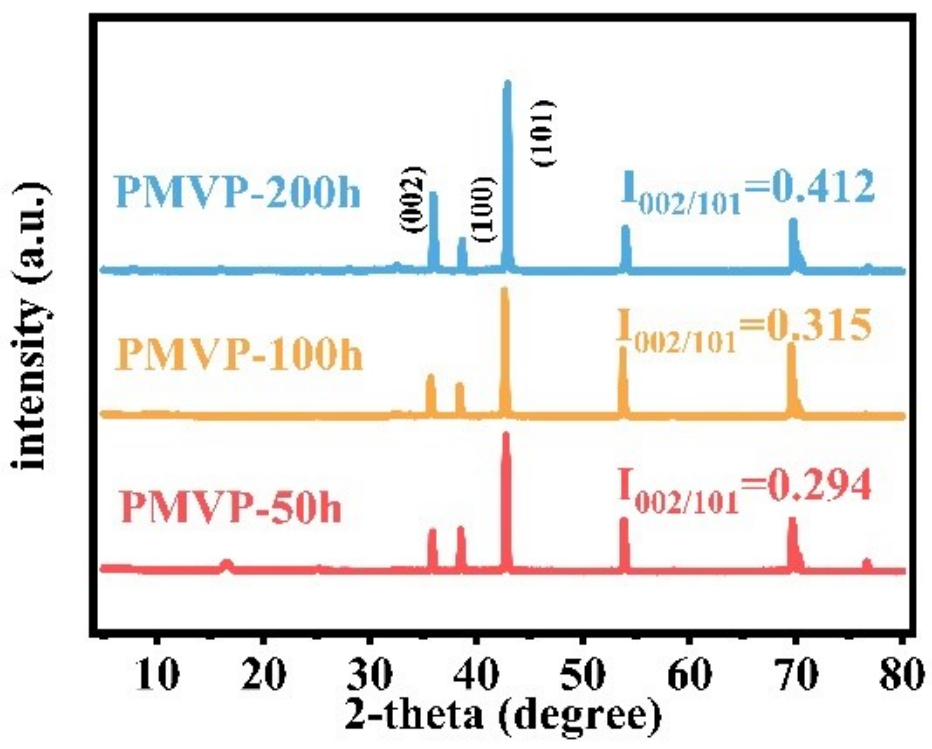

Fig. S17 XRD of Zn anodes after cycling PMVP electrolytes at 1 mA cm^{-2} and 1 mAh cm^{-2} respectively.

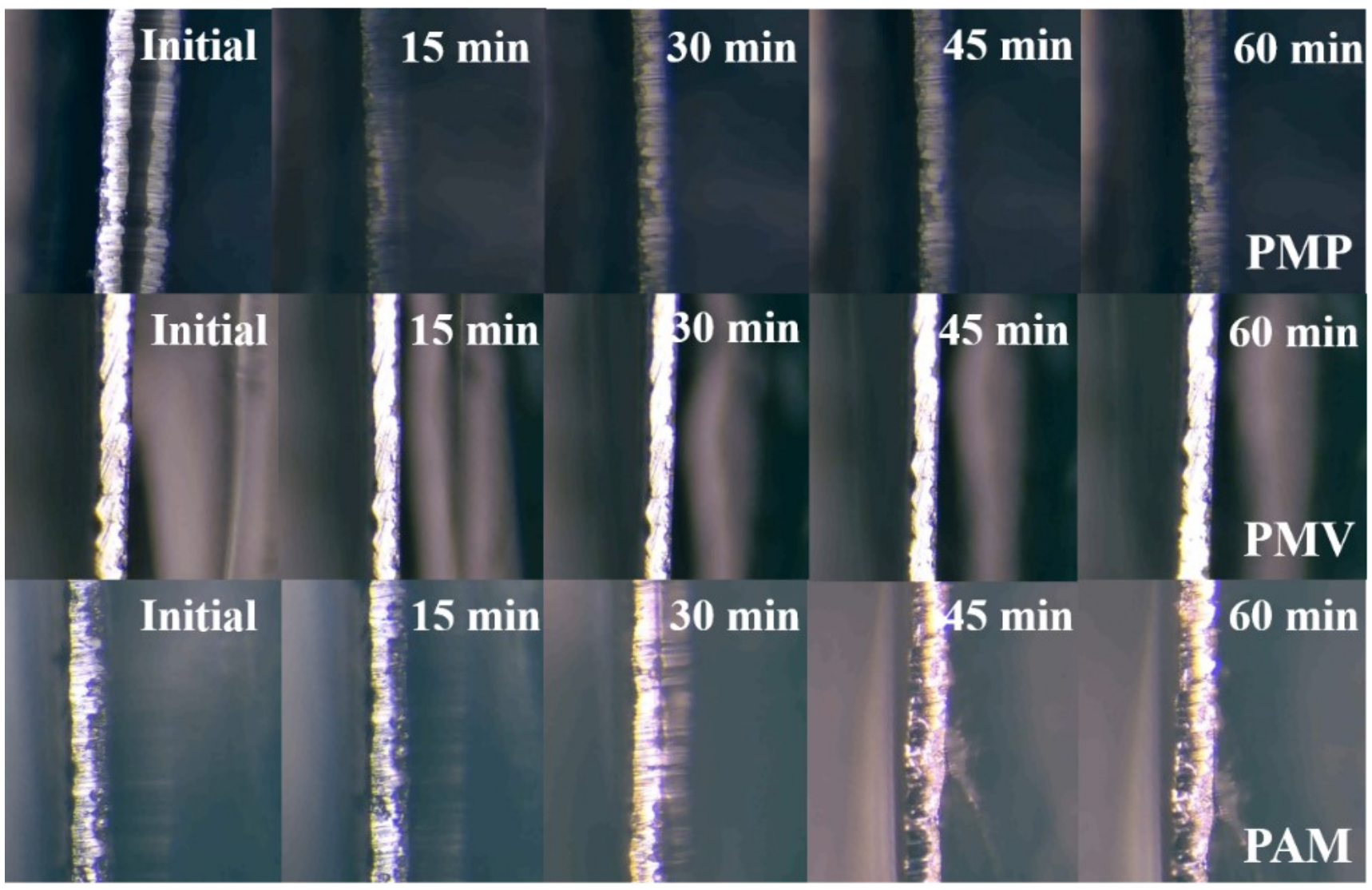

Fig. S18 In-situ optical microscopy images of PMP, PMV and PAM.

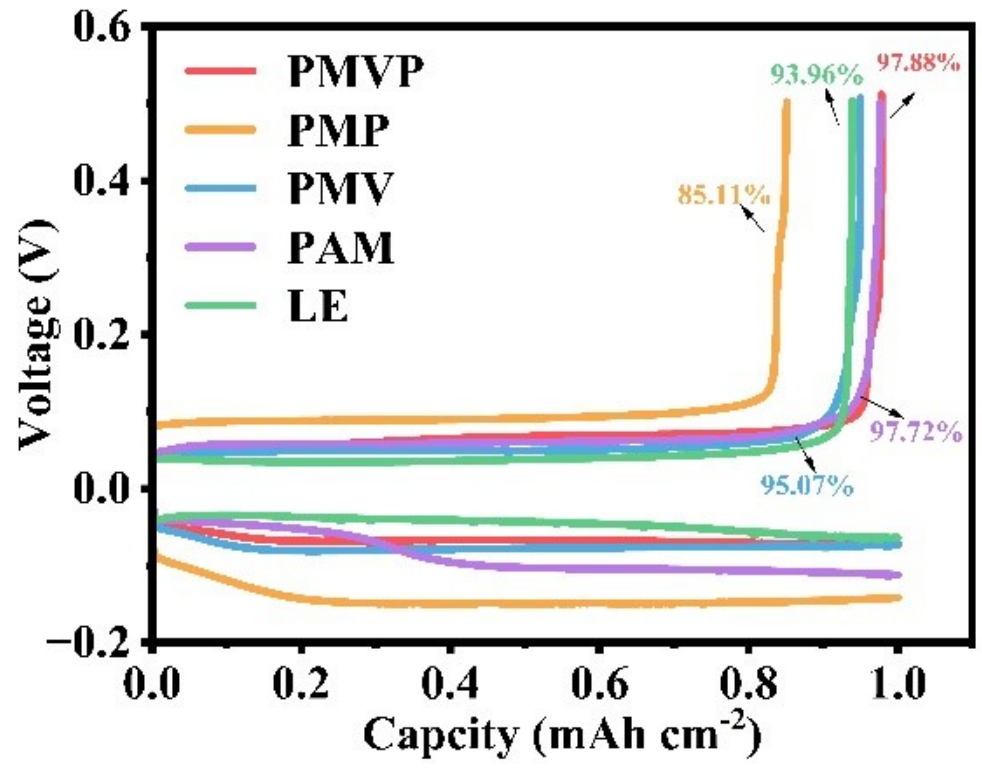

Fig. S19 Voltage curve during the first cycle of the Zn//Cu battery.

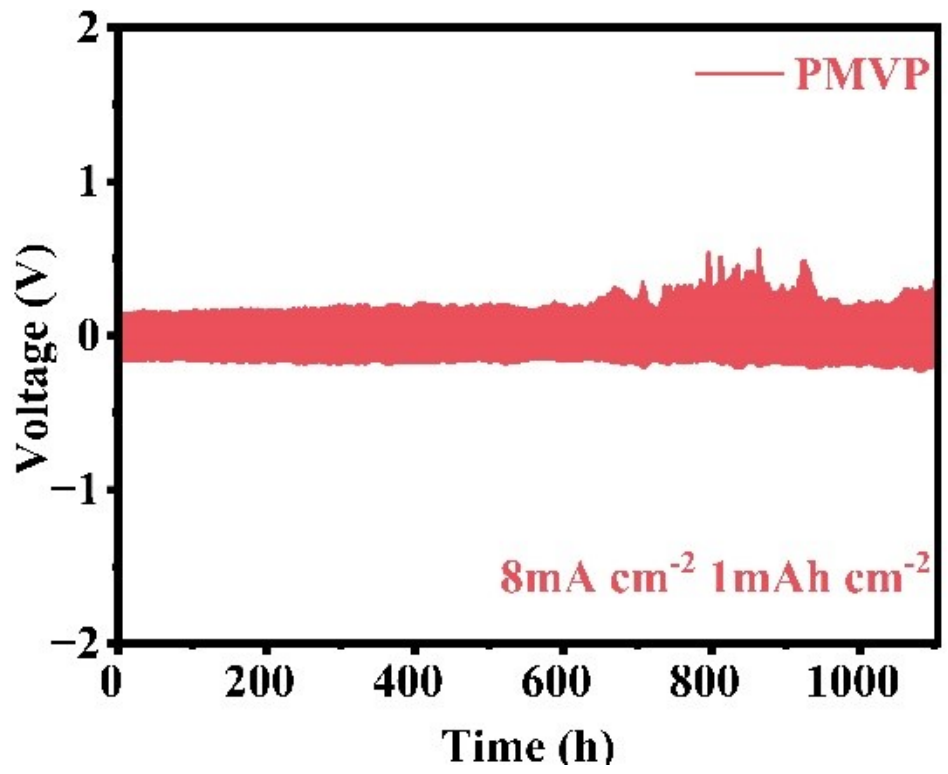

Fig. S20 Cycling performance of the Zn//Zn battery at 8 mA cm^{-2}, 1 mAh cm^{-2}.

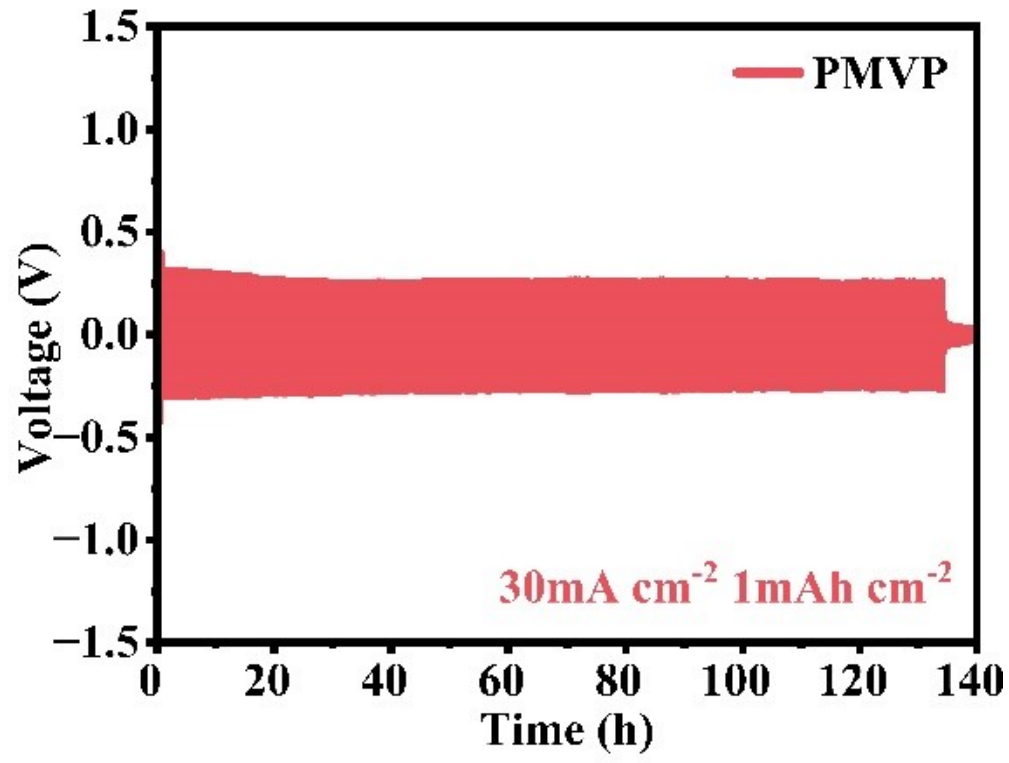

Fig. S21 Cycling performance of the Zn//Zn battery at 30 mA cm^{-2}, 1 mAh cm^{-2}.

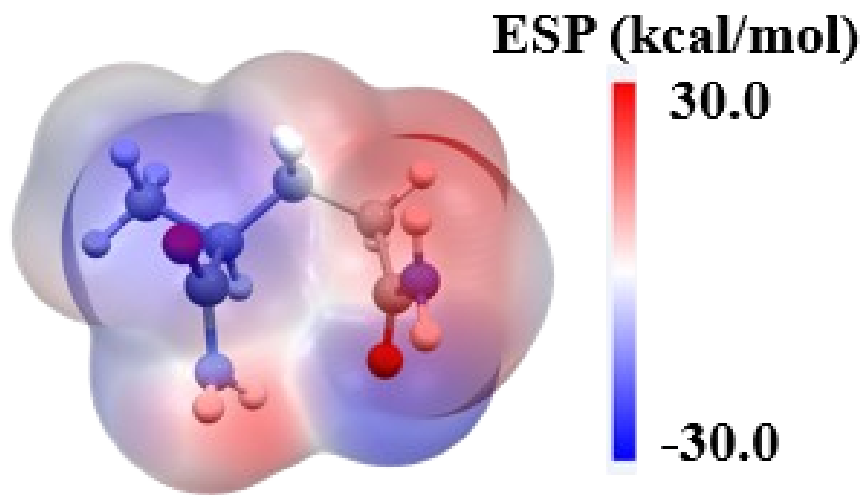

Fig. S22 Electrostatic potential maps of PAM.

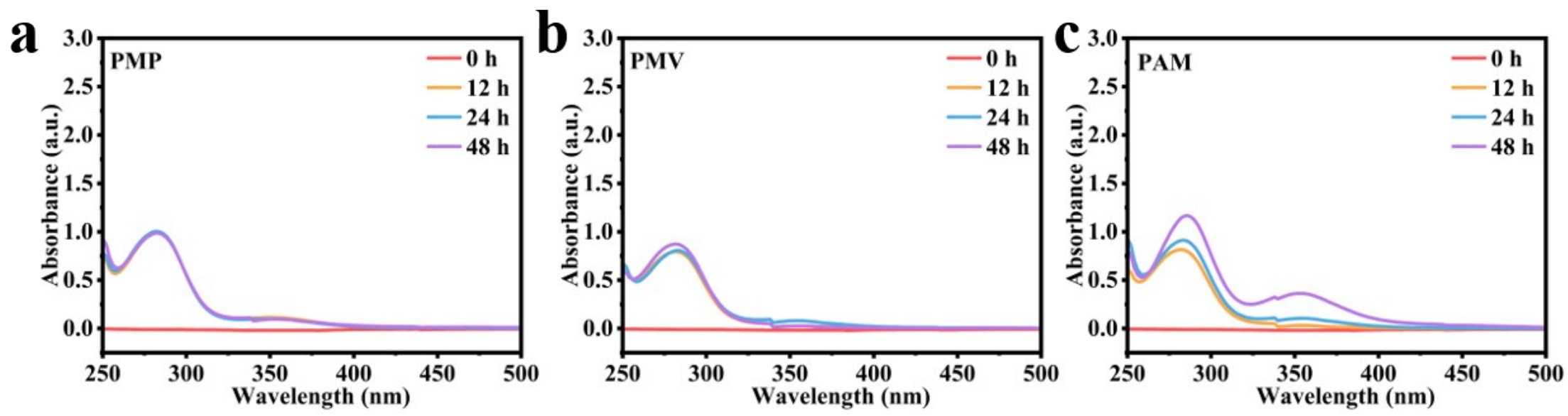

Fig. S23 UV-Vis absorption spectra of the solution in the right chamber for (a) PMP, (b) PMV and (c) PMP separator.

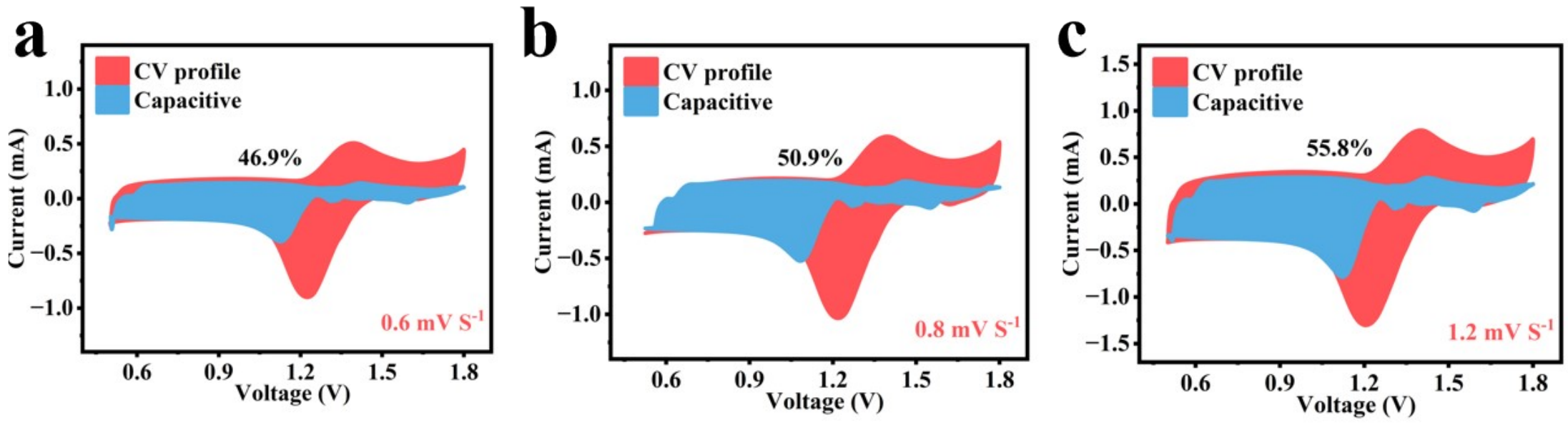

Fig. S24 Fitted capacitive contribution at (a) 0.6 mV S^{-1}, (b) 0.8 mV S^{-1}, (c) 1.2 mV S^{-1} and (d) 0.8 mV S^{-1}.

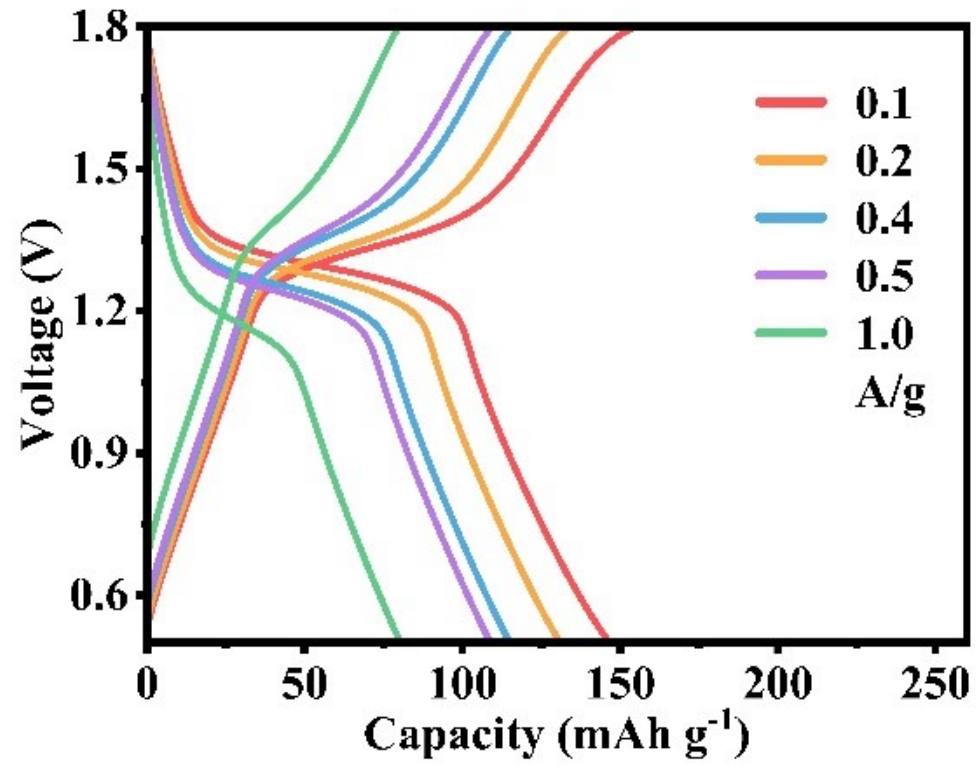

Fig. S25 Voltage-capacity curves of Zn//I₂ batteries with LE electrolyte.

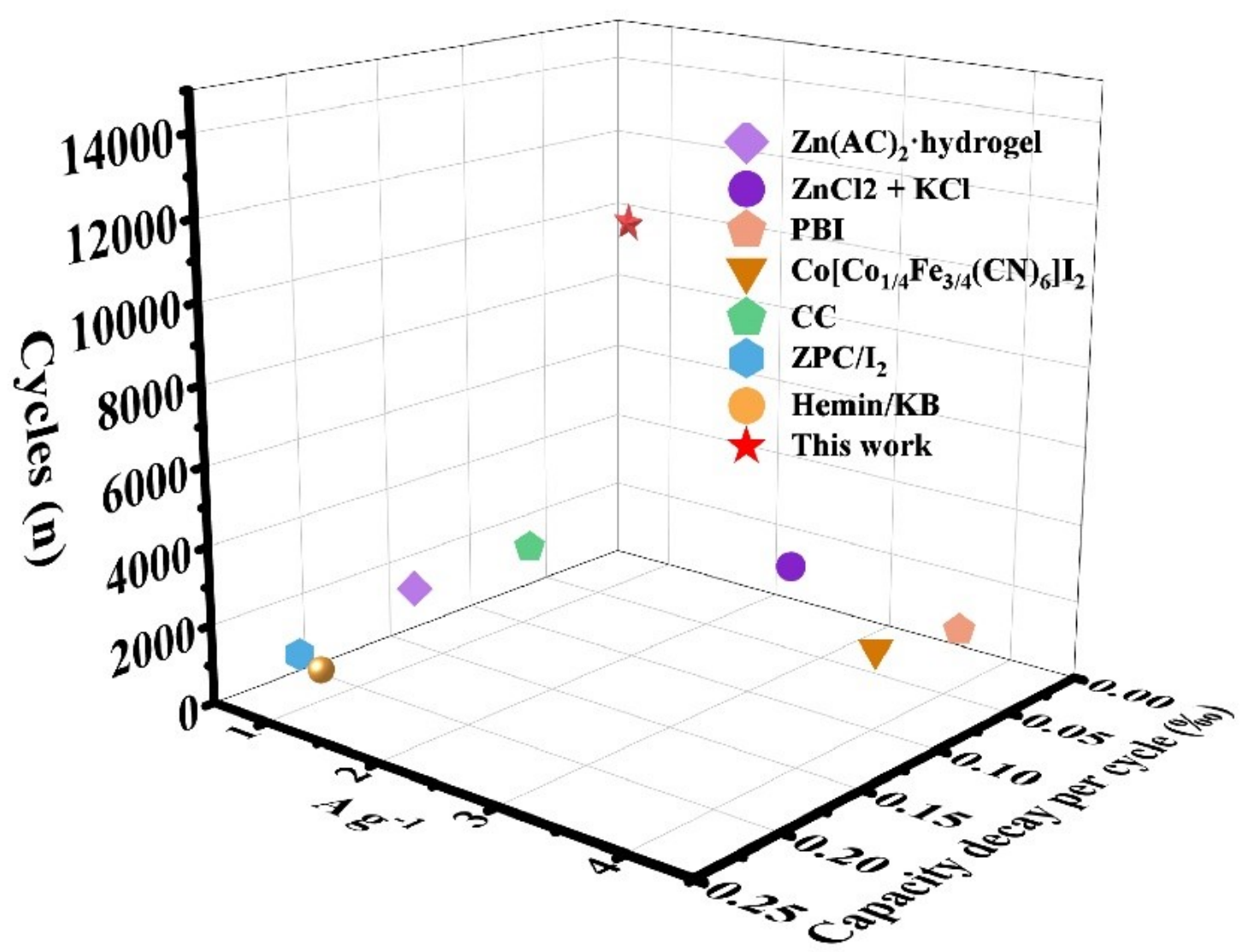

Fig. S26 Comparison of cycle number capacity decay per cycle of this work to reported Zn//I_2 batteries based on designed electrolytes or I_2 cathodes.

Table S1. Molecular composition of PMVP hydrogel electrolyte MD simulation system

Components	PMVP	Na	Br	Zn	TFO
**Number**	14	14	14	100	200
**Molby**	0.122807018	0.122807018	0.122807018	0.877192982	1.754385965
**Average coordination number**	0.063894737	0.122807018	0.032	0.877192982	0.368315789
**Solventation ratio**	52.03%	100.00%	26.06%	100.00%	20.99%
**Free solvent ratio**	47.97%	0.00%	73.94%	0.00%	79.01%

Table S2. Molecular composition of LE hydrogel electrolyte MD simulation system

Components	Zn	TFO	H2O
**Number**	100	200	5555
**Molby**	1	2	55.55
**Average coordination number**	1	0.31684	5.31548
**Solventation ratio**	100.00%	15.84%	9.57%
**Free solvent ratio**	0.00%	84.16%	90.43%

Table S3. The electrochemical performance of Zn//Zn symmetric battery is compared with that of reported hydrogel zinc ion battery.

Modified material	Current density (mA cm^{-2})	Areal Capacity (mAh cm^{-2})	Lifespan (h)	References
PZD	1	1	1600	[5]
CHE	1	1	2850	[6]
HEHE	2	2	1800	[7]
PAM-T-S	1	1	3200	[8]
SCS	1	1	2600	[9]
PAM-DMSO	0.5	0.5	1200	[10]
PAD@SC	0.5	0.5	700	[11]
PDMAPS	1	1	700	[12]
PAM/LA/PSBMA	1	2	681	[13]
Z5S	1	1	2768	[14]
ZSO-2MSA	1	1	1300	[15]
SL	1	1	1300	[16]
$Zn(BF_4)_2$/PC	1	1	1500	[17]
BDO	1	1	1800	[18]
DA	2	1	1000	[19]
**PMVP**	**1**	**1**	**4500**	**This work**

Table S4. Comparison of cycle number capacity decay per cycle of this work to reported Zn//I_2 batteries based on designed electrolytes or I_2 cathodes.

**Electrolyte or I_2 cathode**	**Current density (A g^{-1})**	**Cycles (n)**	**Capacity decay per cycle (‰)**	**References**
$Zn(AC)_2$·hydrogel	1	2000	0.166	[20]
$ZnCl_2$ + KCl	3	2800	0.072	[21]
PBI	4	1500	0.040	[22]
$Co[Co_{1/4}Fe_{3/4}(CN)_6]I_2$	4	2000	0.099	[23]
CC	1	2000	0.095	[24]
ZPC/I_2	0.8	1000	0.22	[25]
Hemin/KB	1	800	0.22	[26]
**PMVP**	**1**	**10000**	**0.029**	**This work**

References

1 S. Izadi and A. V. Onufriev, *Journal of Chemical Physics*, 2016, **145**, 074501.
2 B. Doherty, X. Zhong, S. Gathiaka, B. Li and O. Acevedo, *Journal of Chemical Theory and Computation*, 2017, **13**, 6131.
3 G. Bussi, D. Donadio and M. Parrinello, *Journal of Chemical Physics*, 2007, **126**, 014101.
4 W. Humphrey, A. Dalke and K. Schulten, *Journal of Molecular Graphics & Modelling*, 1996, **14**, 33.
5 J. Zhang, C. Lin, L. Zeng, H. Lin, L. He, F. Xiao, L. Luo, P. Xiong, X. Yang, Q. Chen and Q. Qian, *Small*, 2024, **20**, 2312116.
6 Z. Du, S. Shen, X. Su, Y. Zhuang, M. Chen, X. Zhang, Z. Lin, L. Yu, P. Zhou, M. Wu, X. Lyu and Z. Zou, *Adv Mater*, 2025, **37**, 2502328.
7 Z. Gong, Q. Meng, Y. Zhao, C. Wang, W. Wang and Z. Pei, *Angewandte Chemie International Edition*, 2025, **65**, e23881.
8 J. Guan, Y. Mu, X. Wei, L. Yang, Z. Chen, Q. Man, T. Xue, Y. Li, C. Yang, L. Zang and L. Zeng, *Adv Funct Mater*, 2025, **35**, 2508306.
9 Y. Q. Hu, Z. Wang, Y. Z. Li, P. W. Liu, X. L. Liu, G. X. Liang, D. Zhang, X. Fan, Z. G. Lu and W. X. Wang, *Chem Eng J*, 2024, **479**, 147762.
10 P. Gong, S. Zhai, S. Liu, S. Chai, N. Wang, Y. Li and J. Liu, *Small*, 2025, **22**, 2501089.
11 J. Liu, F. Wang, W. Jiang, Q. Zhao, W. Li, C. Wang, S. Liu and Y. Liu, *Chem Eng J*, 2024, **483**, 149360.
12 S. Zhang, H. Ao, J. Dong, D. Wang, C. Wang, X. Xu, Z. Hou and J. Yang, *Angewandte Chemie International Edition*, 2024, **64**, e202414702.
13 S. Cui, W. Miao, X. Wang, K. Sun, H. Peng and G. Ma, *ACS Nano*, 2024, **18**, 12355.
14 F. F. Wu, J. K. Zhang, L. Ma, P. C. Ruan, Y. L. Chen, S. B. Meng, R. L. Yin, W. H. Shi, W. X. Liu, J. Zhou and X. H. Cao, *Angew Chem-Int Edit*, 2025, **64**, e202421787.
15 X. Y. Wei, Y. B. Mu, J. Chen, Y. K. Zhou, Y. Q. Chu, L. Yang, C. Z. Huang, T. Xue, L. M. Zang, C. Yang and L. Zeng, *Energy Storage Mater*, 2025, **75**, 104026.
16 Z. Y. Peng, L. Tang, S. L. Li, L. C. Tan and Y. W. Chen, *Angew Chem-Int Edit*, 2025, **64**, e202418242.
17 X. X. Guo, K. Y. Zhang, D. L. Han, C. J. Cui, A. N. Liu, Y. Guo, J. C. Gao, R. Sun, C. G. Wei, L. C. Yin, G. J. He, Z. Weng and Q. H. Yang, *Adv Energy Mater*, 2025, **15**.
18 Y. T. Liu, M. J. Qiu, Y. X. Liang, J. H. Zhang, J. G. Chen, P. Sun and W. J. Mai, *Angew Chem-Int Edit*, 2025, **64**, e202506010.
19 Y. Yang, Y. Z. Li, Q. Z. Zhu and B. Xu, *Adv Funct Mater*, 2024, **34**, 2316371.
20 W. S. Shang, J. H. Zhu, Y. Liu, L. T. Kang, S. Y. Liu, B. K. Huang, J. S. Song, X. M. Li, F. Y. Jiang, W. Du, Y. F. Gao and H. J. Luo, *Acs Applied Materials & Interfaces*, 2021, **13**.
21 X. Li, M. Li, Z. Huang, G. Liang, Z. Chen, Q. Yang, Q. Huang and C. Zhi, *Energy Environ Sci*, 2021, **14**.

22 W. Z. Gao, S. T. Cheng, Y. X. Zhang, E. R. Xie and J. C. Fu, *Adv Funct Mater*, 2023, **33**.
23 L. T. Ma, Y. R. Ying, S. M. Chen, Z. D. Huang, X. L. Li, H. T. Huang and C. Y. Zhi, *Angew Chem-Int Edit*, 2021, **60**.
24 L. F. Hang, W. M. Li, T. Zhang and G. H. Jiang, *Chem Eng J*, 2022, **443**, 136230.
25 J. W. Xu, J. G. Wang, L. H. Ge, J. R. Sun, W. Q. Ma, M. M. Ren, X. X. Cai, W. L. Liu and J. S. Yao, *Journal of Colloid and Interface Science*, 2022, **610**, 98-105.
26 Z. H. Chen, F. F. Wang, R. L. Ma, W. Y. Jiao, D. Y. Li, A. Du, Z. J. Yan, T. Y. Yin, X. J. Yin, Q. Li, X. Zhang, N. J. Yang, Z. Zhou, Q. H. Yang and C. P. Yang, *Acs Energy Letters*, 2024, **9**.
